# A new quantum-safe multivariate polynomial public key digital signature algorithm

**DOI:** 10.1038/s41598-022-15843-x

**Published:** 2022-08-01

**Authors:** Randy Kuang, Maria Perepechaenko, Michel Barbeau

**Affiliations:** 1 Quantropi Inc., Ottawa, Ontario K1Z 8P8 Canada; 2 School of Computer Science, Carleton University, Ottawa, K1S 5B6 Canada

**Keywords:** Applied mathematics, Computer science

## Abstract

We propose a new quantum-safe digital signature algorithm called Multivariate Polynomial Public Key Digital Signature (MPPK/DS). The core of the algorithm is based on the modular arithmetic property that for a given element g, greater than equal to two, in a prime Galois field GF(p) and two multivariate polynomials P and Q, if P is equal to Q modulo p-1, then g to the power of P is equal to g to the power of Q modulo p. MPPK/DS is designed to withstand the key-only, chosen-message, and known-message attacks. Most importantly, making secret the element g disfavors quantum computers’ capability to solve the discrete logarithm problem. The security of the MPPK/DS algorithm stems from choosing a prime p associated with the field GF(p), such that p is a sum of a product of an odd prime number q multiplied with a power x of two and one. Given such a choice of a prime, choosing even coefficients of the publicly available polynomials makes it hard to find any private information modulo p-1. Moreover, it makes it exponentially hard to lift the solutions found modulo q to the ring of integers modulo p-1 by properly arranging x and q. However, finding private information modulo the components q and power x of two is an NP-hard problem since it involves solving multivariate equations over the chosen finite field. The time complexity of searching a private key from a public key or signatures is exponential over GF(p). The time complexity of perpetrating a spoofing attack is also exponential for a field GF(p). MPPK/DS can achieve all three NIST security levels with optimized choices of multivariate polynomials and the generalized safe prime p.

## Introduction

The demand for secure communications increased dramatically in the last few decades. Authentication algorithms play a major role in providing security in the digital world. Most of the digital signature algorithms are implemented using the well-known and well-studied cryptosystems Rivest–Shamir–Adleman (RSA)^1^, Digital Signature Algorithm (DSA), and Elliptic Curve Digital Signature Algorithm (ECDSA)^2,3^. However, none of the algorithms based on prime factorization, such as RSA, or the Discrete Logarithm Problem (DLP), such as DSA and ECDSA, are secure against quantum attacks^4^. Thus, a need for a secure Public Key Infrastructure (PKI) created a new effort to find one or efficient quantum-safe digital signature algorithms.

We propose a new quantum-safe digital signature algorithm, Multivariate Polynomial Public Key Digital Signature (MPPK/DS). It stems from the Kuang et al.’s Multivariate Polynomial Public Key (MPPK) Key Encapsulation Mechanism (KEM) algorithm^5^. The MPPK/DS signature scheme design is different from a decryption-encryption digital signature scheme addressing the key-only attack vulnerability, such as with RSA. MPPK/DS also withstand known quantum computing attacks, such as solving DLP, by using a secret random base in modular arithmetic exponentiation in the signing algorithm. In addition, we focus on developing a probabilistic digital signature. The core of the signing-verifying relationship in the MPPK/DS algorithm is a modular arithmetic property that states that given an integer *x* co-prime with *n* and two integers *a* and *b*, if $a = b$ modulo $\varphi (n)$, then $x^a = x^b$ modulo *n*, where $\varphi (n)$ is the Euler’s totient function evaluated at *n*. Most importantly, *a* and *b* are values of multivariate polynomials modulo $\varphi {(p)}$. The security of the algorithm is based on the hardness of solving multivariate polynomials over a large finite field^6^. Moreover, by using a clever choice of prime *p* associated with a finite field ${\mathbb {F}}_p$ that has form $p = 2^xq+1$, where *q* is a large prime, and special choice of the coefficients of the publicly available polynomials, we make it hard for an attacker to find private key components modulo $\varphi (p)$, and exponentially difficult to lift the solutions found modulo *q* and $2^x$ to the ring ${\mathbb {Z}}/\varphi (p){\mathbb {Z}}.$ The best complexity of cracking the MPPK/DS algorithm is ${\mathscr {O}}([2(2\lambda+1)\log p]q^{\frac{3}{2}}2^{x(n+1)+x/2} + 2(\lambda+1)\times 2^x)$ using a classical system, and ${\mathscr {O}}(\sqrt{q2^{x(n+1)}}+(2\lambda+1) \times \sqrt{2^{x}})$ respectively using a quantum device.

We review the related in "Related work". "MPPK digital signature and verification" describes the MPPK/DS algorithm in detail, including key generation, signing, verification, and signing-verifying relationship. Security analysis is done in "Security analysis". We discuss an optimal choice of parameters and give a brief overview of the performance of MPPK/DS in "Brief benchmarking results and optimal parameters of MPPK/DS". The conclusion is drawn in the last section.

## Related work

In the digital age that we live in today, simple day-to-day activities, such as surfing the Internet^7,8^ or performing financial transactions using credit cards^9^, require the use of digital signature cryptosystems. Tan et al. identified 14 real-world applications of digital signature cryptosystems across the financial, critical infrastructure, Internet, and enterprise sectors^3^. Currently, the most widely used digital signature schemes are RSA^1^ and ECDSA^2^. As of July 2013, both are digital signature standards^10^. However, none of them are secure against attacks using fault-tolerant quantum computers^4,11^. That is, ECDSA, like DSA^10^, relies on DLP that can be broken using Shor’s algorithm^4^. RSA depends on the integer factorization problem that can also be efficiently solved using Shor’s algorithm implemented on a quantum computer^4^. Some other digital signature algorithms, such as hash-based algorithms, are considered to be quantum-resistant^12,13^. However, they might not be suitable for specific use-cases, such as when the platform of execution is a chip-card^3^. In an attempt to tackle this issue, National Institute of Standards and Technology (NIST) launched a Post-Quantum Cryptography (PQC) project aiming to standardize one or more key exchange and digital signature algorithms that withstand classical and quantum attacks^14^.

In November 2017, NIST received a total of 82 submissions, out of which seven are digital signature algorithms falling into the category of multivariate algorithms that proceeded to the first round^15^. Only four of the seven first-round candidates moved on to the second round, namely the Lifted Unbalanced Oil and Vinegar (LUOV), Rainbow, GeMSS, and MQDSS schemes. The Rainbow scheme has been accepted as a Round three finalist. GeMSS has been accepted as a Round three alternate candidate. Based on the overall consideration of public key size, signature size, and performance of key generation, signing, and verification, NIST plans to reopen for submissions of PQC digital signature in early 2022.

LUOV^16^ and Rainbow^17^ are both multivariate digital signature algorithms based on the Unbalanced Oil and Vinegar (UOV) scheme, originally introduced by Kipnis et al.^18^. There have been some attacks over the years on the UOV scheme; however, overall, the UOV scheme remains secure^19^. Braeken et al. did a study^20^ of the security of the UOV scheme. They showed that if the number of variables *n* used in the scheme is greater than 2*m*, where *m* is the number of equations used in the scheme, then the cryptosystem is particularly vulnerable to the Gröbner basis attacks. This is an improvement of the result showed by Courtois *et al.* for $n \ge 4m$^21^. Moreover, they showed that choosing coefficients from a small sub-field raises serious security concerns and should be avoided. In addition, Braeken et al. extended the Youssef et al. attack^22^ against Scheme B from Imai and Matsumoto^23^ against the Unbalanced Oil and Vinegar scheme. This new attack is particularly efficient when the number of vinegar variables *v* is small. Faugère and Perret also studied^24^ the security of the UOV scheme. They showed that some of the parameters proposed by Kipnis *et al.*^18^ are not secure against a special Gröbner basis attack. The attack entails computing Gröbner bases of an “easier” system of equations rather than computing a single Gröbner basis for the original system of equations. However, most of these attacks can be resisted by updating the proposed parameters.

Ding et al. developed an attack method on LUOV, called the Subfield Differential Attack (SDA)^25^. SDA does not rely on the Oil and Vinegar structure of LUOV; rather, it takes advantage of the fact that the coefficients of the quadratic terms are contained in a small subfield. This attack reduces the complexity of the LUOV scheme below the targeted security for the NIST post-quantum standardization competition. Ding et al. point out that the SDA does not work on UOV or the Rainbow digital signatures algorithm^25^. Later, Ding et al. proposed a modified SDA, called the Nested Subset Differential Attack, which fully breaks half of the parameter sets of the LUOV scheme and can practically be done in under 210 minutes for the NIST level I security parameters^26^. This attack is the reason for LUOV’s elimination from the standardization project. As the original SDA, the updated Nested SDA does not leverage the UOV scheme and rather takes advantage of the lifting technique of LUOV; thus, it does not apply to the Rainbow digital signature algorithm.

Recall that the Rainbow algorithm relies on the UOV scheme. In addition to the attacks on the UOV scheme described above, Beullens gave two new attacks against the Rainbow signature schemes, namely the intersection and rectangular MinRank attacks^27^. Given the Rainbow third round parameters^17^, these new attacks reduce the cost of a key recovery by a factor of $2^{20}$ for the security level I, $2^{40}$ for the security level III, and $2^{55}$ for security level V, making these parameter sets fall short of the security requirements set out by NIST.

In addition to mathematical cryptanalysis, physical implementation attacks on UOV and Rainbow are also studied to ensure the security of the NIST Round 3 finalists. Hashimoto et al. presented general fault attacks on the multivariate quadratic equations-based schemes^28^. Later, Krämer et al. showed how to apply Hashimoto et al.’s attack to the UOV and Rainbow schemes. It did not, however, lead to the complete private key recovery^29^. Shim et al. performed an extensive fault analysis of UOV and Rainbow^30^. They focused on attacks that cause faults on random Vinegar values used in signing. Shim et al. showed that the equivalent key of UOV is wholly recovered in polynomial time from $(m + 1), n$ and *m* signatures generated by the entire faulty Vinegar values in the three cases, respectively. The equivalent key of Rainbow is also recovered from 44, 79, and 43 signatures with 36 bytes of faulty Vinegar values in the three cases, respectively. This is the first result that leads to the full secret key recovery of UOV and Rainbow from the leakage of the Vinegar values.

Two other NIST round two finalists, the GeMSS^31^ and MQDSS algorithms^32^, have also been studied for any possible security concerns. Kales and Zaverucha presented an attack on the MQDSS scheme^33^. Their attack can be applied to signature schemes built upon five-round identification schemes constructed via the Fiat-Shamir transformation. MQDSS falls under this category. They showed that forging a signature for the 128-bit security level version of MQDSS can be done in $2^{95}$ operations. To avoid the attack, new parameters were proposed that make the scheme significantly worse in performance^15^. That caused the elimination of the MQDSS method from the standardization project. The GeMSS scheme did not have any serious security concerns in Round two. One of the significant drawbacks of the system is enormous public keys, difficulty implementing the algorithm on low-end devices, and slow signing times^19^. The security of the GeMSS scheme relies on HFE construction. Ding et al. studied the security of the HFE cryptosystem^34^. They presented a new algebraic method to attack the HFEv cryptosystem, using the algebraic structure of HFEv. The idea of the attack is to view the new vinegar variables as an external perturbation and to try to separate them, which can be done efficiently for small parameters $D+r$. However, the complexity of the attack is exponential in the small parameter *r*. Overall, the GeMSS scheme is considered secure and is a NIST round-three finalist in the alternative digital signature scheme category.

We now shift the attention to the DLP. The DLP is the core mathematical problem underlying many widely used cryptosystems such as Diffie–Hellman (DH) and Elliptic Curve Diffie–Hellman (ECDH). It is, however, as we pointed out, not secure against attacks using quantum devices^4^. In our digital signature scheme, we use in part a construction similar to the one of DH. However, we do not share the base with the verifier, nor do we share the exponent. Nevertheless, we feel that it is worthwhile investigating any advances related to DLP.

Using Pollard’s rho algorithm one might solve DLP in a cyclic group of size *q* with computational complexity of ${\mathscr {O}}(\sqrt{q})$^35^. Assuming DLP in a group *GF*(*n*), where $n = uv$, if one knows *u* and *v*, one might reduce DLP to a smaller DLP using the Chinese Remainder Theorem and Pohlig–Hellman algorithm. Then it is possible to solve the reduced problem with ${\mathscr {O}}(\sqrt{q})$ modular multiplications^36^.

Boudot et al. set two new records: factorize RSA-240, 795-bit number, and compute a discrete logarithm over a 795-bit prime field. They used the same system to set both records, thus showing that the difficulty of computing discrete logarithm is comparable to the problem of factorization of the same bit size^37^. Granger et al. computed a discrete logarithm in the finite field $GF(2^{30750})$ using the elimination step of the Granger, Kleinjung, and Zumbrägel’s algorithm^38^ recursively. Corrigan-Gibbs and Kogan studied algorithms to solve DLP that utilizes pre-processing^39^. They showed that any generic discrete logarithm algorithm with pre-processed *S*-bit “advice” string runs in online time *T* and succeeds with probability $\epsilon$ if $ST^2 = \Omega (\epsilon N),$ where *N* is the order of the underlying group. They also demonstrated two new generic pre-processing attacks: one for the multiple-discrete-log problem and certain decisional-type problems in groups. Hong et al. proposed a fuzzy Hellman algorithm that solves DLP using a one-time pre-computation process^36^. Given the pre-computation cost and online efficiency, this algorithm performs better than other known algorithms. Bellare introduced the Multi-Base Discrete Logarithm^40^ that fills a gap exhibited by all known standard proofs^41,42^ of the security of Schnorr’s identification and signatures algorithms^43^. Teseleanu produced the first *l* out of *n* threshold kleptographic attack on discrete logarithm-based digital signatures by combining the notions of threshold scheme and kleptographic attack^44^.

Recently, Abdullah et al. presented a new way to solve the elliptic curve DLP, using initial minors^45^. Practical implementation showed that the attack could be performed for groups of orders up to $2^{50}.$

Roetteler et al. gave a precise estimate of quantum resources needed to compute discrete logarithm on elliptic curves over prime fields using Shor’s algorithm^46^. They showed that it takes at most $9n + 2\lceil \log _2(n)\rceil + 10$ qubits to compute discrete logarithm on an elliptic curve defined over *n*-bit prime field, using a quantum circuit of at most $448n^3\log _2(n)+4090n^3$ Toffoli gates. This result supports the one presented earlier by Proos and Zalka^47^ and suggests that the number of qubits required to break Elliptic Curve Cryptography (ECC) is less than the number needed to break RSA. Ekerå bridged their work with Shor’s work on computing discrete logarithms as well as Seifret’s work on computing orders with trade-offs to give an algorithm that computes discrete logarithms without any knowledge of the group order^48^. Moreover, compared to Shor’s algorithm, their algorithm has a factor of two fewer group operations evaluated quantumly in each run, at the expense of multiple runs.

In addition to PQC digital signature schemes, another promising idea using quantum systems to create digital signatures has emerged, called Quantum Digital Signature (QDS). The QDS was first proposed by Gottesman and Chuang^49^, signing classical bits with qubits. QDS offers information-theoretic security of signatures guaranteed by the laws of quantum mechanics. Lü and Feng proposed their QDS based on quantum one-way functions^50^, a novel arbitrated quantum digital signature scheme to sign general quantum states. Clarke et al. experimentally demonstrated QDS using phase-encoded coherent states^51^. Wallen et al. presented their QDS with QKD components and offered their security proof^52^. Hong et al. presented their QDS in a network with a signer, multiple verifiers, and a trusted center, a quantum counterpart of the classical PKI^53^. Single-bit QDS was first extended to multi-bit QDS by Wang et al.^54^ and further by Wang and Wang in 2019 with a more efficient protocol^55^. Inspired by the measurement-device-independent continuous-variable scheme in QKD, Zhao et al. first proposed their Continuous-Variable QDS (CV-QDS) in 2021 for both single-bit and multi-bit schemes^56^. They later improved CV-QDS to remove the loopholes of the practical detectors and eliminate all side-channel attacks^57^.

To visualize the various approaches and differences between the described digital signature schemes, we provide the Table 1. There are two groups of rows: classical data and quantum data. The first column lists classical techniques applicable to classical data. The second group of columns summarizes quantum techniques applicable to classical or quantum data. In the classical data case, we provide the name of the primitive, basis, known most effective attack, and whether it is considered for the NIST third round. In the quantum data case, we provide the name of two applicable techniques.

**Table 1:** Summary of related work.

	Classical technique	Quantum technique			
Classical data		Primitive	Based on	Most effective attack	NIST 3rd round finalist
	RSA^1^	LUOV^16^	UOV^18^	Nested subfield attack^26^	
	DSA	Rainbow^17^	UOV^18^	Min-Rank attacks^27^	$\checkmark$
	ECDSA^2^	GeMSS^31^	HFE^34^	Due to Ding^34^	$\checkmark$
		MQDSS^32^	Fiat-Shamir	Due to Kales and Zaverucha^33^	
Quantum data	–			QDS^49–55^	
				CV-QDS^56,57^	

## MPPK digital signature and verification

MPPK/DS is a digital signature and verification scheme that uses public keys. We formally define the concept of a digital signature and verification using public keys, consistently with other authors^58,59^.

### Definition 3.1

(*Digital signature*) A digital signature scheme is specified by a pair of algorithms. There are two parties: a signer and a verifier. To sign a message $\mu$, the signer uses a signing private key *s* and an algorithm $S_s()$ to create a message-digital signature pair $(\mu ,t)=S_s(\mu )$. The signer sends the pair $(\mu ,t)$. Upon reception of a message-digital signature pair $(\mu ',t')$, the verifier uses a public key *v*, corresponding to *s*, and a signature verifying algorithm $V_v()$ to evaluate if $t'$ is a matching digital signature for $\mu '$. When there is a match, the evaluation $V_v(\mu ')$ returns $(\mu ',VALID)$, otherwise it yields $(\mu ',INVALID)$.

Key generation can also be addressed explicitly. Hence, MPPK/DS comprises three algorithms: key generation, signing and signature verifying. They are respectively described in "Key generation algorithm", "Signing algorithm" and "Signature verifying algorithm".

### Key generation algorithm

MPPK/DS has its own public-key and private-key operations. A key generation algorithm produces a private key and a corresponding public key. Said algorithm is described in this subsection. The algorithm has the following security parameters:

1. A chosen generalized safe prime $p = 2^xq+1$ that determines the index of a finite field defining the domain of all coefficients and variables. Note that its Euler’s totient $\varphi (p)$ is equal to $p-1$, or $2^xq$.
2. Positive integers *m*, *n*,  and $\lambda$, that respectively specify the number of noise variables, the degree of a base polynomial, defined in the Eq. (1), and the degree of two univariate polynomials, defined in the Eq. (3).
3. The positive integers $\ell _1,\ldots ,\ell _m$ that determine the degrees of noise variables in the base polynomial, as defined in Eq. (2).

The signer and verifier agree on the actual values of the security parameters upon establishing communication.

Note that the set *GF*(*p*), or ${\mathbb {Z}}/p{\mathbb {Z}}$. denotes the integers modulo *p*. Let *GF*(*p*) be the domain of variables $x_0,x_1,\ldots ,x_m$. Variable $x_0$ denotes a message or the hashed value of a message. Variables $x_1, \ldots , x_m \ge 1$ represent noise. We also refer to the set $GF(\varphi (p))$, or ${\mathbb {Z}}/\varphi (p){\mathbb {Z}}$, the integers modulo $\varphi (p)$.

With all arithmetic done modulo $\varphi (p)$, the following mathematical objects are created by the signer:

1. A multivariate base polynomial of the form
  1 $$\begin{aligned} \beta (x_0, x_1, \ldots , x_m) = \sum _{i=0}^{n}\sum _{j_1=0}^{l_1}\cdots \sum _{j_m=0}^{l_m}c_{ij_1\ldots j_m}x_0^ix_1^{j_1}\cdots x_m^{j_m}. \end{aligned}$$
  The constants $l_1, \ldots , l_m$ are positive integers. The coefficients $c_{ij_1\ldots j_m}$ are randomly selected from $GF(\varphi (p))$. Written with respect to the variable $x_0$, Eq. (1) is a polynomial of the form
  2 $$\begin{aligned} \beta (x_0, x_1, \ldots , x_m) = \sum _{i = 0}^{n}\beta _i(x_1, \ldots , x_m)x_0^i, \text{ with } \beta _i(x_1, \ldots , x_m) = \sum _{j_1=0}^{l_1}\cdots \sum _{j_m=0}^{l_m} c_{ij_1\ldots j_m} x_1^{j_1}\cdots x_m^{j_m} . \end{aligned}$$
2. Two univariate polynomials of the form
  3 $$\begin{aligned} f(x_0) = \sum _{i=0}^{\lambda } f_i x_0^i \text{ and } h(x_0) = \sum _{i=0}^{\lambda } h_i x_0^i. \end{aligned}$$
  The coefficients $f_i$ and $h_i$ are randomly selected from $GF(\varphi (p))$.
3. Using the base polynomial and two univariate polynomials, two product polynomials are created
  $$\begin{aligned} \phi (x_0, x_1, \ldots , x_m) = f(x_0)\beta (x_0, x_1, \ldots , x_m) \text{ and } \psi (x_0, x_1, \ldots , x_m) = h(x_0)\beta (x_0, x_1, \ldots , x_m). \end{aligned}$$
  Polynomial $\phi (x_0, x_1, \ldots , x_m)$ can also be written in the form (similarly for $\psi (x_0, x_1, \ldots , x_m)$)
  4 $$\begin{aligned} \phi (x_0, x_1, \ldots , x_m) = \sum _{k = 0}^{n+\lambda }\phi _k(x_1, \ldots , x_m)x_0^{k}. \end{aligned}$$
  For $i \in \{0, \ldots , n\}$ and $j \in \{0, \ldots , \lambda \}$, every $\phi _{k}(x_1, \ldots , x_m)$ (similarly for $\psi _{k}(x_1, \ldots , x_m)$) has the form
  5 $$\begin{aligned} \phi _{k}(x_1, \ldots , x_m) = \sum _{k = i+j}f_{i}\beta _{j}(x_1, \ldots , x_m). \end{aligned}$$
4. Two univariate polynomials containing only the message variable $x_0$
  $$\begin{aligned} E_{\phi }(x_0) = \sum _{i = 1}^{n+\lambda -1}e_{{\phi _i}}x_0^i \text{ and } E_{\psi }(x_0) = \sum _{i = 1}^{n+\lambda -1}e_{{\psi _i}}x_0^i \end{aligned}$$
  for randomly selected $e_{\phi _i}, e_{\psi _i} \in GF(\varphi (p))$.
5. Two even random integers $R_0, R_n$ in $GF(\varphi (p))$.
6. Using the integers $R_0, R_n,$ two noise functions are created
  6 $$\begin{aligned} N_0(x_1, \ldots , x_m)= & {} R_0\beta _0(x_1, \ldots , x_m) \text{ and } \end{aligned}$$
  7 $$\begin{aligned} N_n(x_0, x_1, \ldots , x_m)= & {} R_n\beta _{n}(x_1, \ldots , x_m)x_0^{n+\lambda } \end{aligned}$$
  where $\beta _0(x_1, \ldots , x_m)$ and $\beta _n(x_1, \ldots , x_m)$ are as defined in the Eq. (2).
7. Let $\Phi (x_0, x_1, \ldots , x_m)$ be the polynomial $\phi (x_0, x_1, \ldots , x_m)$ without the highest order term and the constant term with respect to the variable $x_0$, namely
  8 $$\begin{aligned} \Phi (x_0,x_1, \ldots , x_m) = \sum _{k = 1}^{n+\lambda -1}\phi _k( x_1, \ldots , x_m)x_0^{k}, \end{aligned}$$
  where the coefficients $\phi _k(x_1, \ldots , x_m)$ are as defined in the Eq. (5), ignoring the constant term and highest order term with respect to the variable $x_0$. Such polynomial is created. Similarly, polynomial
  9 $$\begin{aligned} \Psi (x_0, x_1, \ldots , x_m) = \sum _{k = 1}^{n+\lambda -1}\psi _k(x_1, \ldots , x_m)x_0^{k} \end{aligned}$$
  is created.
8. Using $E_{\phi }(x_0)$, $E_{\psi }(x_0)$, $R_0$, $R_n$, $\Phi (x_0, x_1, \ldots , x_m)$, and $\Psi (x_0, x_1, \ldots , x_m)$ two polynomials
  10 $$\begin{aligned} P(x_0, x_1, \ldots , x_m)= & {} R_0 \left( \Phi (x_0, x_1, \ldots , x_m) - E_{\phi }(x_0) \right) = \sum _{k = 1}^{n+\lambda -1}R_0(\phi _k(x_1, \ldots , x_m)- e_{{\phi _k}})x_0^{k} \text{ and } \end{aligned}$$
  11 $$\begin{aligned} Q(x_0, x_1, \ldots , x_m)= & {} R_n \left( \Psi (x_0, x_1, \ldots , x_m) - E_{\psi }(x_0) \right) = \sum _{k = 1}^{n+\lambda -1}R_n(\psi _k(x_1, \ldots , x_m)- e_{{\psi _k}})x_0^{k} \end{aligned}$$
  are created.

The private key *s* consists of the following items:

1. The two univariate polynomials $f(x_0)$ and $h(x_0)$.
2. The values of the two even noise constants $R_0$ and $R_n$.
3. Polynomials $$E_{\phi }(x_0)$$ and $$E_{\psi }(x_0)$$.

The public key *v* comprises the following elements:

1. The two polynomials $P(x_0, x_1, \ldots , x_m)$ and $Q(x_0, x_1, \ldots , x_m)$.
2. The two noise functions $N_0(x_1, \ldots , x_m)$ and $N_n(x_0, x_1, \ldots , x_m)$.

### Signing algorithm

Let $\mu$ be a message, or the hash of a message. Let $g \in GF(p)$, with $g \ge 2$, be a randomly selected base. All arithmetic is done modulo $\varphi (p)$, unless specified otherwise. Using the signer’s private key *s*, the signing algorithm consists of computing the following items:

1. $$A = g^a \mod p$$, with $$a = R_0f(\mu )$$.
2. $$B = g^b \mod p$$, with $$b = R_nh(\mu )$$.
3. $$C = g^c \mod p$$, with $$c = s_0(\mu ) = R_n[h(\mu )f_0 - f(\mu )h_0]$$.
4. $$D = g^d \mod p$$, with $$d = s_n(\mu ) =R_0[h(\mu )f_{\lambda }-f(\mu )h_{\lambda }]$$.
5. $$E = g^e \mod p$$, with $$e = t(\mu ) = R_0R_n[ h(\mu )E_{\phi }(\mu )-f(\mu )E_{\psi }(\mu ) ]$$.

The digital signature is the quintuple (*A*, *B*, *C*, *D*, *E*). The signing algorithm yields $S_s(\mu ) = (\mu ,(A, B, C, D, E))$. The components *A*, *B*, *C*, *D* and *E* are required to be not equal to 0 or 1. If any of *A*, *B*, *C*, *D* and *E* is 1, a new random base *g* is chosen and a new quintuple (*A*, *B*, *C*, *D*, *E*) is created. The signer sends the pair $((\mu ,(A, B, C, D, E))$ to the verifier.

### Signature verifying algorithm

All arithmetic is done modulo $\varphi (p)$, unless specified otherwise. Upon receiving a message (or the hash of a message) $\mu$ and a corresponding signature (*A*, *B*, *C*, *D*, *E*) from a signer, the verifier applies the signature verifying algorithm using the signer’s public key *v*. Using $\mu$ for $x_0$ and randomly chosen positive values $r_1, \ldots , r_m \in GF(\varphi (p))$ for the noise variables $x_1, \ldots , x_m$, the verifier evaluates the two public polynomials

$$\begin{aligned} {\bar{P}} = P(x_0, x_1, \ldots , x_m) \text{ and } {\bar{Q}} = Q(x_0, x_1, \ldots , x_m). \end{aligned}$$

and the noise functions

$$\begin{aligned} \bar{N_0} = N_0(x_1, \ldots , x_m) \text{ and } \bar{N_n} = N_n(x_0, x_1, \ldots , x_m). \end{aligned}$$

When $A^{{\bar{Q}}}$ is equal to $B^{{\bar{P}}}C^{\bar{N_0}}D^{\bar{N_n}}E$ $\mod p$, the signature verifying algorithm $V_v(\mu )$ returns $(\mu ,VALID)$, otherwise it yields $(\mu ,INVALID)$.

Note that there are multiple choices for the random variables $x_1, \ldots , x_m$. They all produce various values for ${\bar{P}},$ ${\bar{Q}}$, $\bar{N_n}$, and $\bar{N_0}$. Thus, MPPK/DS falls into the category of non deterministic digital signature algorithms in the sense that verifying the same valid signature several times with different values of the polynomial resolves in equalities.

#### Lemma 3.2

(Completeness) Given a message $\mu$, a public key *v* and corresponding private key *s* we have $V_v(S_s(\mu ))=(m,VALID)$.

#### Proof

It follows from the modular arithmetic property: $\text {if }a \equiv b \mod \varphi (p), \text{ then } g^a = g^b \mod p,$ where *g* and *p* are co-prime numbers. Note, that polynomial $R_0R_n\phi (x_0, x_1, \ldots , x_m)$ can be expressed as

12 $$\begin{aligned} R_nf_0N_0(x_1, \ldots , x_m) + R_0f_{\lambda }N_n(x_0, x_1, \ldots , x_m) + R_nP(x_0, x_1, \ldots , x_m) + R_0R_nE_{\phi }(x_0) \end{aligned}$$

and polynomial $R_0R_n\psi (x_0, x_1, \ldots , x_m)$ can be expressed as

13 $$\begin{aligned} R_nh_0N_0(x_1, \ldots , x_m) + R_0h_{\lambda }N_n(x_0, x_1, \ldots , x_m) + R_0Q(x_0, x_1, \ldots , x_m) + R_0R_nE_{\psi }(x_0). \end{aligned}$$

Multiplying polynomial $\phi (x_0, x_1, \ldots , x_m)$ by $R_0R_nh(x_0)$, and $\psi (x_0, x_1, \ldots , x_m)$ by $R_0R_nf(x_0)$ yields the following equality.

14 $$\begin{aligned} R_0R_nh(x_0)\phi (x_0, x_1, \ldots , x_m) = R_0R_nf(x_0)\psi (x_0, x_1, \ldots , x_m). \end{aligned}$$

Using Eqs. (12) and (13), Eq. (14) can be expanded as

15 $$\begin{aligned}{}&h(x_0) \left( R_nf_0N_0(x_1, \ldots , x_m)+ R_0f_{\lambda }N_n(x_0, x_1, \ldots , x_m)+R_nP(x_0, x_1, \ldots , x_m) + R_0R_nE_{\phi }(x_0) \right) = \end{aligned}$$

16 $$\begin{aligned}{}&f(x_0) \left( R_nh_0N_0(x_1, \ldots , x_m)+ R_0h_{\lambda }N_n(x_0, x_1, \ldots , x_m)+R_0Q(x_0, x_1, \ldots , x_m) + R_0R_nE_{\psi }(x_0) \right) . \end{aligned}$$

This expression can be rewritten as

17 $$\begin{aligned} f(x_0)R_0Q(x_0, x_1, \ldots , x_m)=\, & {} h(x_0)R_nP(x_0, x_1, \ldots , x_m)+N_0(x_1, \ldots , x_m)s_0(x_0)\nonumber \\&+ N_n(x_0, x_1, \ldots , x_m)s_n(x_0) + t(x_0). \end{aligned}$$

where $s_0(x_0) = R_n(h(x_0)f_0 - f(x_0)h_0$), $s_n(x_0) = R_0(h(x_0)f_{\lambda } - f(x_0)h_{\lambda })$, and $t(x_0) = R_0R_n \left( h(x_0)E_{\phi }(x_0) - f(x_0)E_{\psi }(x_0) \right) .$ Performing modular exponentiation of Eq. (17) results in

$$\begin{aligned} g^{f(x_0)R_0Q(x_0, x_1, \ldots , x_m)} = g^{h(x_0)R_nP(x_0, x_1, \ldots , x_m)+N_0(x_1, \ldots , x_m)s_0(x_0) + N_n(x_0, x_1, \ldots , x_m)s_n(x_0) + t(x_0)} \text { mod }p. \end{aligned}$$

This equality can be rewritten as

$$\begin{aligned} g^{a{\bar{Q}}} = g^{b{\bar{P}}} g^{c\bar{N_0}} g^{d\bar{N_n}} g^{e} \mod p \text{ or } A^{{\bar{Q}}} = B^{{\bar{P}}} C^{\bar{N_0}} D^{\bar{N_n}} E \mod p. \end{aligned}$$

## Security analysis

We discuss attack models on digital signature algorithms. There are three attack types: chosen-message, known-message, and key-only. There are two sub-categories in the chosen-message attack: direct-chosen and generic-chosen, depending on whether the adversary knows the public key. If the adversary knows the public key, then the direct-chosen method can replace a message signed by the signer with a message the adversary wants but with the signer’s signature. If the adversary does not know the public key, then the generic-chosen method can trick the signer into digitally signing a message that it does not intend to sign. In the known message attack, the adversary obtains old messages and signatures. It tries to forge signatures for messages that the signer does not intend to sign. It uses brute force to analyze old data to recreate the signer’s signature. This attack is analogous to the known-plaintext attack on encryption. The signer’s public key is assumed to be available to everyone in the key-only attack. The adversary uses this fact and tries to recreate the signer’s signature and digitally sign messages that the signer does not intend to do. This causes a significant threat to the authentication of messages, which is non-repudiated as the signer cannot deny signing it.

A digital signature using RSA^1^, without hashing messages, is vulnerable to the known-message and chosen-message attacks. This is due to its multiplicative property where a product of messages leads to a product of their signatures. Once an attacker knows the public key, then the signer is requested to sign a public key encrypted message *y*. The returning signature *x* forms a message-signature pair of *x* and *y* called a key-only attack. Therefore, the RSA digital signature must be used with a cryptographic hash function. The ElGamal digital signature^60^ and Digital Signature Algorithm (DSA)^10^, based on the DLP, also require the use of a cryptographic message hash function to prevent existential forgery.

These digital signature attacks are not applicable to MPPK/DS. Unlike the RSA signature scheme, MPPK/DS is not a one-way trap door type of digital signature, with decryption for signing and encryption for verification. It is also not a DLP-type signature scheme like DSA, with a public generator as modulo arithmetic exponentiation base. Most importantly, it does not use a secret message directly as the exponent in the modulo arithmetic exponentiation to calculate the signature. It uses polynomials evaluated at the message in the exponent for modulo arithmetic exponentiation. Therefore, MPPK/DS is not vulnerable to the above signature attacks. Furthermore, techniques for solving the DLP, such as the ones using the Shor’s quantum algorithm, are not directly applicable.

Cracking MPPK/DS boils down to producing a signature for a fake message that passes verification. In other words, it requires a universal or selective forgery of signatures. To achieve that, adversaries must crack public keys or signatures to obtain private keys or directly brute force the values *A*, *B*, *C*, *D*, and *E* consistently with the verification relationship. In the remainder of this section, we analyze the security of MPPK/DS. We examine possible approaches a malicious party could take to obtain the private key from a public key and a signature. We also discuss digital signature spoofing vulnerabilities.

### Security of the private key given the public key

MPPK/DS stems from MPPK KEM. Most of the security analysis done by Kuang et al.^5^ directly applies to MPPK/DS. Public keys in both algorithms are almost identical, except for the modulo $\varphi (p)$ in the exponent polynomial computations. They share the same relationship with the corresponding private keys.

We start by considering whether it is possible to obtain any components of the private key from the published coefficients of polynomials $P(x_0, x_1, \ldots , x_m)$ and $Q(x_0, x_1, \ldots , x_m).$ Note that all the calculations involving private and public keys are performed modulo $\varphi (p) = p-1 = 2^xq.$ Recall, that every term of the coefficients of the public polynomials $P(x_0, x_1, \ldots , x_m)$ and $Q(x_0, x_1, \ldots , x_m)$ contains $R_0$ and $R_n$ respectively. Since both $R_0$ and $R_n$ are not co-prime with $\varphi (p)$, it is not possible for a malicious party to solve the system of equations generated by the coefficients of $P(x_0, x_1, \ldots , x_m)$ and $Q(x_0, x_1, \ldots , x_m)$ correctly in the ring ${\mathbb {Z}}/\varphi (p){\mathbb {Z}}.$ That is because it is impossible to divide by the terms containing $R_0$ and $R_n$ in the ring ${\mathbb {Z}}/\varphi (p){\mathbb {Z}}.$ However, *q* and $2^x$ are co-prime, so the ring of integers ${\mathbb {Z}}/\varphi (p){\mathbb {Z}}\cong {\mathbb {Z}}/q{\mathbb {Z}}\times {\mathbb {Z}}/2^x{\mathbb {Z}}.$ Hence, calculations to obtain private keys from public keys can essentially be performed modulo *q* and $2^x,$ and then lifted to modulo $\varphi (p) = 2^xq.$ Notice, that since $R_0$ and $R_n$ are even it is not possible to gain any information modulo $2^x$. Hence, the attacker is reduced to solving the system of equations modulo *q*, and lifting the solutions to the ring ${\mathbb {Z}}/\varphi (p){\mathbb {Z}}$ in order to find the actual solution.

Since it is not possible to fully solve the system of equations generated by the coefficients of $P(x_0, x_1, \ldots , x_m)$ and $Q(x_0, x_1, \ldots , x_m)$ modulo $\varphi (p)$ or $2^x$, since $R_0$ and $R_n$ are even, we turn our attention to the ring ${\mathbb {Z}}/q{\mathbb {Z}}$. We first discuss two ways of considering the publicly available coefficients of $P(x_0, x_1, \ldots , x_m)$ and $Q(x_0, x_1, \ldots , x_m)$ modulo *q*. One way is to consider the coefficients of $P(x_0, x_1, \ldots , x_m)$ and $Q(x_0, x_1, \ldots , x_m)$ that are not associated with the pure $x_0$ term, namely $p_{kj_1j_2\ldots j_m}$ and $q_{kj_1j_2\ldots j_m}$ for all $k \in \{1, \ldots , n+\lambda -1\}$ and $j_1j_2\ldots j_m \ne 00\ldots 0.$ Solving this system of equations does not give the attacker any information about the signature component *E*. The other way for a malicious party to find private keys from public keys is to consider all of the shared coefficients of $P(x_0, x_1, \ldots , x_m)$ and $Q(x_0, x_1, \ldots , x_m)$ including coefficients $p_{k00\ldots 0} = \sum _{k = t+s}f_tc_{s00\ldots 0}-e_{\phi _{k}}$ and $q_{k00\ldots 0} = \sum _{k = t+s}h_tc_{s00\ldots 0}-e_{\psi _{k}}$ respectively for all $k \in \{1, \ldots , n+\lambda -1\}.$ The latter approach involves systems of equations with more variables and equations. Note also that the term *E* can be derived from *A*, *B*, *C*, and *D* as $E = A^QB^{-P}C^{-N_0}D^{-N_n}.$

We start by considering the first approach, namely the one without coefficients of $P(x_0, x_1, \ldots , x_m)$ and $Q(x_0, x_1, \ldots , x_m)$ associated with pure $x_0$ terms modulo *q*. Note that similar to MPPK KEM^5^, we can set the public key parameters in such a way that the attacker is faced with an underdetermined systems of equations when considering the shared coefficients of the polynomial $P(x_0, x_1, \ldots , x_m)$ separately from the shared coefficients of the polynomial $Q(x_0, x_1, \ldots , x_m).$

#### Proposition 4.1

Let $$\beta (x_0, x_1, \ldots , x_m) = \sum _{i=0}^{n}\sum \limits _{\begin{subarray}{c} j_k=0\\ k \in \{1, \ldots m\} \end{subarray}}^{l_k}c_{ij_1 \ldots j_m} x_{0}^{i} x_1^{j_1}\ldots x_m^{j_m}$$ be the base polynomial. Publicly available coefficients of $P(x_0, x_1, \ldots , x_m)$, without pure $x_0$ terms, form an underdetermined system of equations, when $(\lambda -2)([\prod _{k=1}^{m}(l_k+1)]-1) < \lambda +2$. The same holds true for the coefficients of $Q(x_0, x_1, \ldots , x_m)$ considered independently without the pure $x_0$ terms.

#### Proof

Let $(\lambda -2)([\prod _{k=1}^{m}(l_k+1)]-1) < \lambda +2$. Publicly available coefficients of $P(x_0, x_1, \ldots , x_m)$ or $Q(x_0, x_1, \ldots , x_m)$ considered independently, and without pure $x_0$ terms, form a system of

$$\begin{aligned} (n+\lambda -1)\left( \left[ \prod _{k=1}^{m}(l_k+1)\right] -1\right) \ \text { equations in } \left[ (n+1)\left( \left[ \prod _{k=1}^{m}(l_k+1)\right] -1\right) \right] +\lambda +2 \ \text { variables, } \end{aligned}$$

if the base polynomial is defined as $$\beta (x_0, x_1, \ldots , x_m) = \sum _{i=0}^{n}\sum \limits _{\begin{array}{c} j_k=0\\ k \in \{1, \ldots m\} \end{array}}^{l_k}c_{ij_1 \ldots j_m} x_{0}^{i} x_1^{j_1}\ldots x_m^{j_m}.$$ Since $(\lambda -2)([\prod _{k=1}^{m}(l_k+1)]-1) < \lambda +2$, the number of variables $\left[ (n+1)\left( \left[ \prod _{k=1}^{m}(l_k+1)\right] -1\right) \right] +\lambda +2$ is greater than the number of equations $(n+\lambda -1)\left( \left[ \prod _{k=1}^{m}(l_k+1)\right] -1\right) .$

#### Corollary 4.2

Let the base polynomial be $\beta (x_0, x_1, \ldots , x_m) = \sum _{i = 0}^{n}\left( \sum _{j=1}^{J} c_{ij}X_j\right) x_0^i,$ where $X_j = \prod _k x_k^{j_k}$ for any desired $j_k$. Then publicly available coefficients of $P(x_0, x_1, \ldots , x_m)$, without pure $x_0$ terms, form an underdetermined system of equations, when $(\lambda -2)(J-1) < \lambda +2$. The same holds true for publicly available coefficients of $Q(x_0, x_1, \ldots , x_m)$ considered independently without the pure $x_0$ terms.

#### Proof

Let the base polynomial be defined as $\beta (x_0, x_1, \ldots , x_m) = \sum _{i = 0}^{n}\left( \sum _{j=1}^{J} c_{ij}X_j\right) x_0^i,$ where $X_j = \prod _k x_k^{j_k}$ for any desired $j_k$. In this case, the coefficients of the polynomial $P(x_0, x_1, \ldots , x_m)$ or $Q(x_0, x_1, \ldots , x_m)$ without pure $x_0$ terms considered independently from one another form a system of

$$\begin{aligned} (n+\lambda -1)(J-1) \ \text { equations in } (n+1)(J-1)+\lambda +2 \ \text { variables. } \end{aligned}$$

Let $(\lambda -2)(J-1) < \lambda +2$. Then, the number of variables $(n+1)(J-1)+\lambda +2$ is greater than the number of equations $(n+\lambda -1)(J-1).$

#### Proposition 4.3

When the coefficients of $P(x_0, x_1, \ldots , x_m)$ and $Q(x_0, x_1, \ldots , x_m)$, without the pure $x_0$ terms, are examined together they form an overdetermined system of equations.

#### Proof

Let the base polynomial be as defined in the Corollary 4.2. Considering two public polynomials $P(x_0, x_1, \ldots , x_m)$ and $Q(x_0, x_1, \ldots , x_m)$ together yields a system of

$$\begin{aligned} 2(n+\lambda -1)(J-1) \ \text { equations in } (n+1)(J-1) + 2\lambda + 4 \ \text { unknowns. } \end{aligned}$$

Equivalently, if the base polynomial is defined as in the Proposition 4.1, then considering $P(x_0, x_1, \ldots , x_m)$ together with $Q(x_0, x_1, \ldots , x_m)$ yields a system of

$$\begin{aligned} 2(n+\lambda -1)\left( \left[ \prod _{k=1}^{m}(l_k+1)\right] -1\right) \ \text { equations in } (n+1)\left( \left[ \prod _{k=1}^{m}(l_k+1)\right] -1\right) + 2\lambda + 4 \ \text { unknowns. } \end{aligned}$$

We now consider the second approach, namely the one that includes coefficients of $P(x_0, x_1, \ldots , x_m)$ and $Q(x_0, x_1, \ldots , x_m)$ associated with pure $x_0$ term modulo *q*.

#### Proposition 4.4

Let the base polynomial be $$\beta (x_0, x_1, \ldots , x_m) = \sum _{i=0}^{n}\sum \limits _{\begin{subarray}{c} j_k=0\\ k \in \{1, \ldots m\} \end{subarray}}^{l_k}c_{ij_1 \ldots j_m} x_{0}^{i} x_1^{j_1}\ldots x_m^{j_m}$$. Let $(\lambda -2)(\prod _{k=1}^{m}(l_k+1)) < n+2\lambda +1$. The shared coefficients of the polynomial $P(x_0, x_1, \ldots , x_m)$, including the pure $x_0$ terms, considered separately from the shared coefficients of the polynomial $Q(x_0, x_1, \ldots , x_m)$, and vice versa, produce an underdetermined system of equations.

#### Proof

Let the base polynomial be defined as $$\beta (x_0, x_1, \ldots , x_m) = \sum _{i=0}^{n}\sum \limits _{\begin{subarray}{c} j_k=0\\ k \in \{1, \ldots m\} \end{subarray}}^{l_k}c_{ij_1 \ldots j_m} x_{0}^{i} x_1^{j_1}\ldots x_m^{j_m}$$. Considering the shared coefficients of the polynomial $P(x_0, x_1, \ldots , x_m)$ separately from the shared coefficients of the polynomial $Q(x_0, x_1, \ldots , x_m)$ and vice versa produces a system of

$$\begin{aligned} (n+\lambda -1)\left( \prod _{k=1}^{m}(l_k+1)\right) \ \text { equations in } \left[ (n+1)\left( \prod _{k=1}^{m}(l_k+1)\right) \right] +n+ 2\lambda +1 \ \text { variables. } \end{aligned}$$

Let $(\lambda -2)(\prod _{k=1}^{m}(l_k+1)) < n+2\lambda +1$. The number of variables $\left[ (n+1)\left( \prod _{k=1}^{m}(l_k+1)\right) \right] +n+ 2\lambda +1$ is greater than the number of equations $(n+\lambda -1)\left( \prod _{k=1}^{m}(l_k+1)\right)$.

#### Corollary 4.5

Let the base polynomial be defined as $\beta (x_0, x_1, \ldots , x_m) = \sum _{i = 0}^{n}\left( \sum _{j=1}^{J} c_{ij}X_j\right) x_0^i,$ where $X_j = \prod _k x_k^{j_k}$ for any desired $j_k$. Let $(\lambda -2)J < n+2\lambda +1$. The shared coefficients of the polynomial $P(x_0, x_1, \ldots , x_m)$, including the pure $x_0$ terms, considered separately from the shared coefficients of the polynomial $Q(x_0, x_1, \ldots , x_m)$, and vice versa, produce an underdetermined system of equations.

#### Proof

Let the base polynomial be $\beta (x_0, x_1, \ldots , x_m) = \sum _{i = 0}^{n}\left( \sum _{j=1}^{J} c_{ij}X_j\right) x_0^i,$ where $X_j = \prod _k x_k^{j_k}$ for any desired *k* and $j_k$. Considering all the public coefficients of $P(x_0, x_1, \ldots , x_m)$ or $Q(x_0, x_1, \ldots , x_m)$ separately produces a system of

$$\begin{aligned} (n+\lambda -1)J \ \text { equations in } \left[ (n+1)J\right] +n+ 2\lambda +1 \ \text { variables. } \end{aligned}$$

Let $(\lambda -2)J < n+2\lambda +1$. Then such system is underdetermined.

#### Proposition 4.6

If the publicly available coefficients of the polynomials $P(x_0, x_1, \ldots , x_m)$ and $Q(x_0, x_1, \ldots , x_m)$ are considered together, they can produce an overdetermined or an underdetermined system of equations, depending on the parameters $n, \lambda , m,$ and $l_k$ for each $k \in \{1, \ldots , m\}$.

#### Proof

Let the base polynomial be as in the Proposition 4.1. Considering the coefficients of $P(x_0, x_1, \ldots , x_m)$ and $Q(x_0, x_1, \ldots , x_m)$ together, they will produce a systems of

$$\begin{aligned} 2(n+\lambda -1)\left( \prod _{k=1}^{m}(l_k+1)\right) \ \text { equations in } \left[ (n+1)\left( \prod _{k=1}^{m}(l_k+1)\right) \right] +2n+ 4\lambda +2 \ \text { variables. } \end{aligned}$$

Then if $\lambda = 3, n = 2,$ and $\prod _{k=1}^{m}(l_k+1) = 5$, the system of equations produces by the coefficients of $P(x_0, x_1, \ldots , x_m)$ together with $Q(x_0, x_1, \ldots , x_m)$ is overdetermined. On the other hand, if $n = 2, \lambda = 2,$ and $\prod _{k=1}^{m}(l_k+1) = 3$, such system is underdetermined.

Equivalently, if the base polynomial is defined as in the Proposition 4.2, then public polynomials considered together result in the system of

$$\begin{aligned} 2(n+\lambda -1)J \ \text { equations in } \left[ (n+1)J\right] +2n+ 4\lambda +2 \ \text { variables. } \end{aligned}$$

Such system is overdetermined if $\lambda = 3, n = 2,$ and $J = 5$, and underdetermined when $n = 2, \lambda = 2,$ and $J = 3$.

We claim that one possible way for the attacker to solve the systems of equations produced by the coefficients of the shared polynomials $$P(x_0, x_1, \ldots , x_m)$$ and $$Q(x_0, x_1, \ldots , x_m)$$, regardless of whether it is underdetermined or overdetermined, is to solve the system modulo *q* first, then lift the solutions to the ring $${\mathbb {Z}}/\varphi (p){\mathbb {Z}}.$$ For instance, assume that the attacker can solve the system of equations produced by the polynomial $$P(x_0, x_1, \ldots , x_m)$$ in the ring $${\mathbb {Z}}/q{\mathbb {Z}}$$ to find $$f_0$$. This result considered modulo $$\varphi (p)$$ is not a single value, but rather an entire equivalence class or equivalently a list of values of the form $$f_0 + iq$$ less than $$\varphi (p)$$ for positive integers *i*. Such a list consists of $$2^x$$ values. One of the list values is the correct solution modulo $$\varphi (p).$$ One way to deterministically conclude whether the value is correct is to solve the same system of equations in the ring $${\mathbb {Z}}/2^x{\mathbb {Z}}$$. Similarly, consider the equivalence class generated by the solution $$f_0$$ modulo $$2^x$$ to lift it to the ring $${\mathbb {Z}}/\varphi (p){\mathbb {Z}}$$. The correct value modulo $$\varphi (p)$$ is an element present in both equivalence classes or lists. On its own, this problem depends on the size of the lists, or equivalently the number of elements of the equivalence classes less than $$\varphi (p)$$. Note, however, that the attacker is unable to fully solve the system of equations modulo $$2^x$$, since $$R_0$$ and $$R_n$$ are even numbers, thus it is impossible to find an inverse of $$R_0$$ or $$R_n$$ in the ring $${\mathbb {Z}}/2^x{\mathbb {Z}}$$. So the attacker is reduced to only solving the system of equations in the field $${\mathbb {Z}}/q{\mathbb {Z}}$$, and then trying to lift the solution to the ring $${\mathbb {Z}}/\varphi (p){\mathbb {Z}}$$ using another way. The complexity of solving underdetermined systems of *m* equations in *n* unknowns over a field $${\mathbb {F}}_{q}$$ is $${\mathscr {O}}\left( q^{m-\min \left( \frac{m}{2}, \left\lfloor \sqrt{\frac{n}{2}-\sqrt{\frac{n}{2}}}\right\rfloor \right) }\right)$$^21^. The complexity of solving an overdetermined system of equations modulo *q* is $${\mathscr {O}}\left( q^{n}\left( \frac{2.718k}{\log q}\right) 
^{n}\right)$$, where *n* is the number of variables, and *k* is the highest degree of the polynomials^61^. Note that the results found modulo *q* are not deterministic, since the lifting step adds uncertainty to the solution. One way to successfully lift the solutions modulo *q* to modulo $$\varphi (p)$$ is to recreate the terms of the form $$p_{kj_1j_2\ldots j_m}$$ and $$q_{kj_1j_2\ldots j_m}$$, where $$p_{kj_1j_2\ldots j_m}$$ and $$q_{kj_1j_2\ldots j_m}$$ are coefficients of the polynomials $$P(x_0, x_1, \ldots , x_m)$$ and $$Q(x_0, x_1, \ldots , x_m)$$ respectively using the elements in the equivalence classes of the solutions found modulo *q*. Classical complexity of this lifting approach is $${\mathscr {O}}\left( 2c\left\lceil \frac{n+1}{\lambda +1}\right\rceil 2^{xv}\right) ,$$ where *v* is the number of unknowns, and *c* is an integer that depends on *n*, and $$\lambda$$. Quantum complexity is $${\mathscr {O}}\left( \sqrt{2c\left\lceil \frac{n+1}{\lambda +1}\right\rceil 2^{xv}}\right)$$ due to Grover’s algorithm. Depending on if the attacker is including the pure $$x_0$$ terms, *v* and *c* will vary.

Thus, as with MPPK KEM^5^, the malicious party chooses whether to take advantage of the shared coefficients and solve an overdetermined system of equations or consider an underdetermined system of equations and use the solution to such system to solve another set of equations. Let $p_{kj_1\ldots j_m}$ be the shared coefficient of the polynomial $P(x_0, x_1, \ldots , x_m)$ associated with the term $x_0^kx_1^{j_1}\cdots x_m^{j_m}$.

#### Claim 4.7

There exists a way to attack the publicly available coefficients of $P(x_0, x_1, \ldots , x_m)$ and $Q(x_0, x_1, \ldots , x_m)$ modulo *q*, and then lift the solution to the ring ${\mathbb {Z}}/\varphi (p){\mathbb {Z}}$. This attack has classical complexity of ${\mathscr {O}}\left( q^{\lambda +2}\left[ 2\left\lceil \frac{n+1}{\lambda +2}\right\rceil 2^{x(\lambda +2)}\right] \right) .$

#### Proof

Begin by working in the ring ${\mathbb {Z}}/q{\mathbb {Z}}$. First, use brute force search to find the noise coefficient $R_0$. Then, divide the values of the shared coefficients $p_{k11\ldots 1}$ by $R_0$ to obtain new values $p'_{k11\ldots 1}$, for all $k \in \{1, \ldots , n+\lambda -1\}$. Suppose that $\lambda = 3$. The system of equations generated by the new coefficients of public polynomials can be viewed as

$$\begin{aligned} \underbrace{\begin{bmatrix} f_1 &{} f_0 &{} 0 &{} 0 &{} 0 &{} 0 &{} \cdots &{} 0 &{} 0 \\ f_2 &{} f_1 &{} f_0 &{} 0 &{} 0 &{} 0 &{} \cdots &{} 0 &{} 0 \\ f_3 &{} f_2 &{} f_1 &{} f_0 &{} 0 &{} 0 &{} \cdots &{} 0 &{} 0 \\ 0 &{} f_3 &{} f_2 &{} f_1 &{} f_0 &{} 0 &{} \cdots &{} 0 &{} 0 \\ 0 &{} 0 &{} f_3 &{} f_2 &{} f_1 &{} f_0 &{} \cdots &{} 0 &{} 0 \\ \vdots &{} \vdots &{} \vdots &{} \vdots &{} \vdots &{} \vdots &{} \vdots &{} \vdots &{} \vdots \\ 0 &{} 0 &{} 0 &{} \cdots &{} 0 &{} 0 &{} \cdots &{} f_2 &{} f_1 \\ \end{bmatrix}}_{M}\begin{bmatrix} c_{011\ldots 1}\\ c_{111\ldots 1}\\ c_{211\ldots 1}\\ \vdots \\ c_{n11\ldots 1}\\ 0\\ 0 \end{bmatrix} = \begin{bmatrix} p'_{111\ldots 1}\\ p'_{211\ldots 1}\\ p'_{311\ldots 1}\\ p'_{411\ldots 1}\\ p'_{511\ldots 1}\\ \vdots \\ p'_{(n+2)11\ldots 1}. \end{bmatrix} \end{aligned}$$

Then the coefficients $c_{ij_1\ldots j_m}$ of the base polynomial can be expressed as

$$\begin{aligned} \begin{bmatrix} c_{011\ldots 1}\\ c_{111\ldots 1}\\ c_{211\ldots 1}\\ c_{311\ldots 1}\\ \vdots \\ c_{n11\ldots 1}\\ 0 \end{bmatrix} = M^{-1}\begin{bmatrix} p'_{111\ldots 1}\\ p'_{211\ldots 1}\\ p'_{311\ldots 1}\\ p'_{411\ldots 1}\\ p'_{511\ldots 1}\\ \vdots \\ p'_{(n+2)11\ldots 1}. \end{bmatrix} \end{aligned}$$

The values $p'_{k11\ldots 1}$ are known for each $k \in \{1, \ldots , n+\lambda -1\}$. Suppose that the values for $f_t$ are found for each $t \in \{0, 1, \ldots , \lambda \}$, then it is possible to find the coefficients $c_{i11\ldots 11}$ for all $i \in \{0, \ldots , n\}$. Once the coefficients $c_{i11\ldots 11}$ are found, they can be directly substituted in the system of equation generated by the publicly available terms $q_{k11\ldots 1}$ of $Q(x_0, x_1, \ldots , x_m)$ to solve for $h_t' = R_nh_{t}$ for all $t \in \{0, 1, \ldots , \lambda \}$. Suppose that $R_n$ is known, then it is a simple calculation to find $h_t$ for all $t \in \{0, \ldots , \lambda \}.$ Note that the attacker can divide coefficients of $N_n$ by the coefficients of the base polynomial to derive $R_n$. Then the attacker can construct signature components *A*, *B*, *C*, and *D* once they lift the solutions to the ring ${\mathbb {Z}}/\varphi (p){\mathbb {Z}}$. The malicious party can use values *A*, *B*, *C* and *D* to derive *E* since $E = A^QB^{-P}C^{-N_0}D^{-N_n}$. So in order to find all the private information modulo *q* necessary to forge a signature one needs to brute force search $R_0$ and $f_t$ for all $t \in \{0, \ldots , \lambda \}$. The complexity of this part of the approach is ${\mathscr {O}}\left( q^{\lambda +2}\right)$ using classical system and ${\mathscr {O}}\left( \sqrt{q^{\lambda +2}}\right)$ using a quantum system.

Note however, that to find the original value modulo $\varphi (p)$, the attacker needs to lift the solutions modulo *q* to modulo $\varphi (p)$. The attacker knows actual shared values of the coefficients of $P(x_0, x_1, \ldots , x_m)$ and $Q(x_0, x_1, \ldots , x_m)$. Thus, the attacker can try to recreate these coefficients using elements of the equivalence classes or lists generated by the solutions modulo *q* to find a match between the actual value and the one recreated by the attacker. The classical complexity of the lifting method is ${\mathscr {O}}\left( 2\left\lceil \frac{n+1}{\lambda +2}\right\rceil 2^{x(\lambda +2)}\right) .$ Using Grover’s algorithm, quantum complexity of the lifting method is ${\mathscr {O}}\left( \sqrt{2\left\lceil \frac{n+1}{\lambda +2}\right\rceil 2^{x(\lambda +2)}}\right) .$

Overall, the classical complexity of this attack is ${\mathscr {O}}\left( q^{\lambda +2}\left[ 2\left\lceil \frac{n+1}{\lambda +2}\right\rceil 2^{x(\lambda +2)}\right] \right)$ and the quantum complexity of this attack is ${\mathscr {O}}\left( \sqrt{q^{\lambda +2}\left[ 2\left\lceil \frac{n+1}{\lambda +2}\right\rceil 2^{x(\lambda +2)}\right] }\right)$.

It is worth mentioning, that in the case of digital signatures, there exists a way to simplify some of the equations produced by the coefficients of $P(x_0, x_1, \ldots , x_m)$ and $Q(x_0, x_1, \ldots , x_m)$. Let $p_{ij_1\ldots j_m}$ be a coefficient of the polynomial $P(x_0, x_1, \ldots , x_m)$ associated with the term $x_0^ix_1^{j_1}\cdots x_m^{j_m}$. Since the coefficients of the noise function $N_0(x_1, \ldots , x_m)$ are components of the terms $p_{lj_1j_2\ldots j_m}$ for $l \in \{1, \ldots , \lambda \}$ and $j_k$; $k \in \{1, 2, \ldots , m\}$, their values can be directly substituted in these expressions. Similar calculations can be done for the coefficients of $N_n(x_0, x_1, \ldots , x_n)$ and terms $q_{kj_1j_2\ldots j_m}$ for $j_l$; $l \in \{1, 2, \ldots , m\},$ and $k \in \{n , n+1, \ldots , n+\lambda -1\}.$ Such advantage does not effect the solution modulo $\varphi (p)$, since $R_0$ and $R_n$ are not co-prime to $\varphi (p),$ however these substitutions do benefit the attacker working in the ring ${\mathbb {Z}}/q{\mathbb {Z}}$ by providing unique solution modulo *q*. Lifting the solutions up to the ring ${\mathbb {Z}}/\varphi (p){\mathbb {Z}}$ has complexity ${\mathscr {O}}(2c(2^{xv})),$ where *v* is the number of unknowns and *c* is some constant that depends on $\lambda$ and *n*.

Another attack on the public keys is described in the Kuang’s *et al.’s* MPPK KEM paper. It leverages the fact that the malicious party can produce as many noise functions $N_0$ and $N_n$ as they want, and solve the system produced by the noise variables to retrieve private information. However, similarly to MPPK KEM, if the malicious party generated a set of equations of the form $N_0(x_0, x'_1, \ldots , x'_m) = \bar{N_0}$ aiming to find $R_0$ or the coefficients of the form $c_{0j_1\ldots j_m}$, they are unable to succeed. In the MPPK/DS case the inability to carry out this attack comes from the incapacity to divide by $R_0$, since $R_0$ is not co-prime to $\varphi (p)$. The same holds true for equations of the form $\bar{N_n} = N_n(x_0, x'_1, \ldots , x'_m)$, and $R_n$ not co-prime with $\varphi (p).$ If the attacker considers these equations modulo *q*, they have the same issue as we described in Kuang *et al.*’s paper^5^, namely the system will produce all zero results.

One of the differences between the MPPK KEM and MPPK/DS algorithms is that the ratios of the form $\frac{f_0}{h_0}, \frac{f_\lambda }{h_\lambda }$, $\frac{c_{0j_1\ldots j_m}}{c_{0j'_1\ldots j'_m}}$, and $\frac{c_{nj_1\ldots j_m}}{c_{nj'_1\ldots j'_m}}$ for any $j_1\ldots j_m$ cannot be derived modulo $\varphi (p)$ in the MPPK/DS algorithm since $R_0$, and $R_n$ are not co-prime with $\varphi (p)$. This makes the MPPK/DS algorithm more secure in the sense that it is not possible to obtain explicit relationships between the components of the private key.

We now describe another attack on the public key carried out in the ring $GF(\varphi (p))$. Considering only the public key, one strategy for the attack in the ring ${\mathbb {Z}}/\varphi (p){\mathbb {Z}}$ is to brute force search for the terms $R_0, R_n$, $f_t$, and $h_t$ for all $t \in \{0, \ldots , \lambda \}$ in the ring ${\mathbb {Z}}/\varphi (p){\mathbb {Z}}$. The complexity of this search is ${\mathscr {O}}([(\varphi (p))^{2\lambda +2}][\varphi (p)-\varphi (p-1)]^{2})$ using classical device and ${\mathscr {O}}([\sqrt{(\varphi (p))^{2\lambda +2}]}[\varphi (p)-\varphi (p-1)])$ using a quantum system. Given the values for $R_0, R_n$, $f_t$, and $h_t$ for all $t \in \{0, \ldots , \lambda \}$, the attacker can produce the signature components *A*, *B*, *C*, and *D* for any hashed message $x_0$. The malicious party can use values *A*, *B*, *C* and *D* to derive *E* since $E = A^QB^{-P}C^{-N_0}D^{-N_n}$, thus, fully forge the signature.

However, the next attack on the public key in the ring ${\mathbb {Z}}/\varphi (p){\mathbb {Z}}$ is far more efficient.

#### Claim 4.8

Finding the private key from public key in the ring ${\mathbb {Z}}/\varphi (p){\mathbb {Z}}$ has an optimal complexity of ${\mathscr {O}}([\varphi (p)]^{n+1}+ 2 \times 2^{x(\lambda+1)})$, classically, and ${\mathscr {O}}(\sqrt{[\varphi (p)]^{n+1}}+ 2 \times \sqrt{2^{x(\lambda+1)}})$ using a quantum computer.

#### Proof

For the sake of simplicity, let us suppose that $\lambda = 3$. Begin by brute force searching for values $c_{i111\ldots 1}$ for all $i \in \{0, 1, \ldots , n\}.$ The complexity of this step is ${\mathscr {O}}(\varphi (p)^{n+1})$ classically and ${\mathscr {O}}(\sqrt{\varphi (p)^{n+1}})$ using quantum computer. The coefficients $p_{k11\ldots 1}$ of a public key polynomial $P(x_0, \dots, x_m)$ for $k \in \{1, \ldots , n+\lambda -1\}$ can be expressed as

$$\begin{aligned} \underbrace{ \begin{bmatrix} c_{111\ldots 1} &{} c_{011\ldots 0} &{} 0 &{} 0 &{} \cdots &{} 0 \\ c_{211\ldots 1} &{} c_{111\ldots 1} &{} c_{011\ldots 1} &{} 0 &{} \cdots &{} 0 \\ c_{311\ldots 1} &{} c_{211\ldots 1} &{} c_{111\ldots 1} &{} c_{011\ldots 1} &{} \cdots &{} 0 \\ c_{4111\ldots 1} &{} c_{3111\ldots 1} &{} c_{211\ldots 1} &{} c_{111\ldots 1} &{} \cdots &{} 0 \\ c_{5111\ldots 1} &{} c_{4111\ldots 1} &{} c_{3111\ldots 1} &{} c_{2111\ldots 1} &{}\cdots &{} 0 \\ \vdots &{} \vdots &{} \vdots &{} \vdots &{} \vdots &{} \vdots \\ 0 &{} 0 &{} c_{n111\ldots 1} &{} c_{(n-1)111\ldots 1} &{} \cdots &{} c_{1111\ldots 1} \\ \end{bmatrix} }_{M} \begin{bmatrix} f_0'\\ f_1'\\ f_2'\\ f_3'\\ 0\\ \vdots \\ 0 \end{bmatrix} = \begin{bmatrix} p_{111\ldots 1}\\ p_{211\ldots 1}\\ p_{311\ldots 1}\\ p_{411\ldots 1}\\ p_{511\ldots 1}\\ \vdots \\ p_{(n+2)11\ldots 1} \end{bmatrix}, \end{aligned}$$

where $f'_t = R_0f_t$ for all $t \in \{0, 1, \ldots , \lambda \}.$ Then the variables $f'_t$ can be found using

$$\begin{aligned} \begin{bmatrix} f_0'\\ f_1'\\ f_2'\\ f_3'\\ 0\\ \vdots \\ 0 \end{bmatrix} = M^{-1}\begin{bmatrix} p'_{111\ldots 1}\\ p'_{211\ldots 1}\\ p'_{311\ldots 1}\\ p'_{411\ldots 1}\\ p'_{511\ldots 1}\\ \vdots \\ p'_{(n+2)11\ldots 1} \end{bmatrix}. \end{aligned}$$

Equivalent calculations can be done for the variables $$h'_t = R_nh_t$$ for all $$t \in \{0, 1, \ldots , \lambda \}.$$ The attacker can first verify if the coefficients $$c_{i11\ldots 1}$$ found using brute force search are correct. For that, the attacker can check if all $$f'_t$$s are zero for $$t >\lambda$$. If the condition is met, then verify if all $$h'_t$$s are zero for $$t >\lambda$$. Then we have a candidate list of $$f'_t$$, $$h'_t$$, and $$c_{i11\ldots 1}$$ for $$i\in \{0, 1, \ldots , n\}$$. If the list only contains a single set of those coefficients, we then find the right coefficients. Having this information, the attacker can create signature components *A* and *B*. In order to create *C* and *D*, the attacker needs to find $$f_i$$ and $$h_i$$ for all *i ∈ {*0*, ..., λ}*. The most efficient way to do that would be to find it modulo *q* and then lift it to the ring $${\mathbb {Z}}/\varphi (p){\mathbb {Z}}.$$ The attacker knows $$R_0f_0, c_{111\ldots 11}$$ and $$N_0$$ modulo *q*, these values can be used to find $$f_i$$ modulo *q* for all *i ∈ {*0*, ..., λ}*. Similar calculations are done for the values *h*_0_, ..., *h*_λ_ in the field *GF(q).* The adversary then needs to lift these values to the ring $$\mathbb {Z}$$/φ(p)$$\mathbb {Z}$$. Classical complexity of this part is $$\mathscr {O}(2^{x(\lambda+1)})$$ because the adversary needs to test that the lifting is successful by confirming that  $$\frac{R_0f_i}{f_i} = R_0$$ for all *i* ∈ {0, ..., λ}. Same is true for values of *h*_0_, ..., *h*_λ_. Using Grover's algorithm implemented on a quantum device, the complexity becomes $${\mathscr {O}}(2 \times \sqrt{2^{x(\lambda+1)}})$$ for all 2(λ+1) values. Now the attacker is lacking only the signature component *E*, which he can get through *A*, *B*, *C*, *D* since $$E = A^Q
B^{-P}C^{-N_0}D^{-N_n}$$. Hence, the overall complexity of this attack is $${\mathscr {O}}([\varphi (p)]^{n+1}+ 2 \times 2^{x(\lambda+1)})$$ using classical system and $${\mathscr {O}}(\sqrt{[\varphi (p)]^{n+1}}+ 2 \times \sqrt{2^{x(\lambda+1)}})$$ using quantum system.

#### Corollary 4.9

Given the public key, the most efficient attack for the private key has classical complexity of ${\mathscr {O}}(\varphi (p)^{n+1}+2 \times 2^{x(\lambda+1)})$ and quantum complexity of ${\mathscr {O}}(\sqrt{\varphi (p)^{n+1}}+2 \times \sqrt{2^{x(\lambda+1)}})$.

### Security of the private key given the signature

As mentioned in "Key generation algorithm", neither the base *g* nor the exponents $R_0f(x_0), R_nh(x_0), s_0(x_0), s_n(x_0)$ or $t(x_0)$ are known to anyone but the signing party. The signer simply shares the signature *A*, *B*, *C*, *D* and *E*. We now examine whether there are relationships between the signature components that a malicious party can exploit.

#### Proposition 4.10

There is no explicit way to express *A* and *B* in terms of each other in the ring ${\mathbb {Z}}/\varphi (p){\mathbb {Z}}$.

#### Proof

Recall, that $A = g^{R_0f(x_0)}$ and $B = g^{R_nh(x_0)}$, where $R_0f(x_0), R_nh(x_0)$ are calculated modulo $\varphi (p) = 2^xq$. If we consider the definition of a logarithm as $\log _AB$ is a constant *t*, such that $A^t =B$ mod p, it is apparent that $t = \frac{h(x_0)R_n}{f(x_0)R_0}.$ However, the element $\frac{1}{R_0}$ does not exist modulo $\varphi (p)$, since $R_0$ is not co-prime with $\varphi (p).$ Thus, such value *t* cannot be computed modulo $\varphi (p).$ Similarly, $\log _BA = \frac{f(x_0)R_0}{h(x_0)R_n}$ but $\frac{1}{R_n}$ does not exist in the ring ${\mathbb {Z}}/\varphi (p){\mathbb {Z}}$. Hence, there is no explicit way to express *A* and *B* in terms of each other in the ring ${\mathbb {Z}}/\varphi (p){\mathbb {Z}}$.

These values exist, however, modulo *q*. The attacker might be able to calculate them to find a ratio of the form $\frac{h(x_0)R_n}{f(x_0)R_0}$ modulo *q*. It will not be possible, however, to correctly lift this value to the ring ${\mathbb {Z}}/\varphi (p){\mathbb {Z}}$ since the solution modulo $2^x$ does not exist. However, if we consider the signature together with the public key there is a way to find public key, and as a result, forge the signature. We discuss this approach later, towards the end of the section. Moreover, if the adversary uses Shor’s algorithm to solve for a discrete logarithm, he will run into a problem. Indeed, let ${\bar{g}}$ be a generator of a multiplicative group $({\mathbb {Z}}/p{\mathbb {Z}})^{\times }$, then

$$\begin{aligned} \log _{{\bar{g}}}A = \log _{{\bar{g}}}g^{R_0f(x_0)} = R_0f(x_0) \times \log _{{\bar{g}}}g \text { (mod }\varphi (p)), \end{aligned}$$

where none of the terms $g, R_0,$ or $f(x_0)$ are known. Therefore, given the numerical value of $\log _{{\bar{g}}}A$ it is not be possible to conclude anything about the private key. Similarly,

$$\begin{aligned} \log _{{\bar{g}}}B = \log _{{\bar{g}}}g^{R_nh(x_0)} = R_nh(x_0) \times \log _{{\bar{g}}}g \text { (mod }\varphi (p)), \end{aligned}$$

where $R_n, h(x_0),$ and *g* are unknown. Thus, taking discrete logarithms of values *A* and *B* does not yield any explicit information modulo $\varphi (p).$ Considering these logarithms modulo *q*, is the same as $\log _BA$ since $\log _BA = \frac{\log _{{\bar{g}}}A}{\log _{{\bar{g}}}B}.$

#### Proposition 4.11

There is no explicit way for the elements *C* and *D* to be expressed in terms of *A* and *B* modulo $\varphi (p)$

#### Proof

Consider

$$\begin{aligned} C = g^{R_n[h(x_0)f_0-f(x_0)h_0]} = B^{f_0} \times A^{-\frac{R_n}{R_0}h_0} \text { (mod }p). \end{aligned}$$

The expression $\frac{R_n}{R_0}$ does not exist mod $\varphi (p)$. So it is not be possible to express *C* in terms of *A* and *B* modulo $\varphi (p)$. Similarly, *D* could be written as

$$\begin{aligned} D = B^{\frac{R_0}{R_n}f_{\lambda }} \times A^{-h_{\lambda }} \text { (mod }p) \end{aligned}$$

but $\frac{R_0}{R_n}$ does not exist mod $\varphi (p)$. Taking discrete logarithm does not yield any meaningful information either since

$$\begin{aligned} \log _{{\bar{g}}}C = f_0 \times \log _{{\bar{g}}}B + \left( -\frac{R_n}{R_0}h_0\right) \times \log _{{\bar{g}}}A \text { (mod }\varphi (p)), \end{aligned}$$

where $f_0, h_0$ are unknown, and $\frac{R_n}{R_0}$ does not exist modulo $\varphi (p).$ For the same reasons $\log _{{\bar{g}}}D$ does not offer any meaningful information.

On the other hand, note that the expression

$$\begin{aligned} R_0\log _{{\bar{g}}}C = R_0f_0\log _{{\bar{g}}}B - R_nh_0\log _{{\bar{g}}}A \text { (mod }\varphi (p)), \end{aligned}$$

where multiplication by $R_0$ is purely symbolic, can exist modulo $\varphi (p)$. Then one might suggest to create a system of such equations for different values of *A*, *B*, and *C* in order to find $R_0, R_n, h_0,$ and $f_0.$ Note, however, that it is not possible to solve such system as it will not be possible to express one variable in terms of the other. Indeed, expressing $f_0$ or $h_0$ in terms of $R_n$ or $R_0$ requires dividing by $R_0$ and $R_n$ respectively. Expressing $R_0$ and $R_n$ in terms of other values requires dividing by $\log _{{\bar{g}}}B$ or $\log _{{\bar{g}}}A$, however, both of these values are a multiple of $R_n$ and $R_0$ respectively, thus, not co-prime to $\varphi (p).$ So approaching the problem this way does not provide a solution to the attacker. Similar argument can be made for $R_n\log _{{\bar{g}}}D = R_nf_{\lambda }\log _{{\bar{g}}}B - R_0h_{\lambda }\log _{{\bar{g}}}A.$

Nevertheless, these expressions can be considered modulo *q*, but it is exponentially hard to lift the solution to ${\mathbb {Z}}/\varphi (p){\mathbb {Z}}$. Note that systems of equations with polynomials such as $R_0\log _{{\bar{g}}}C = R_0f_0\log _{{\bar{g}}}B - R_nh_0\log _{{\bar{g}}}A$ considered modulo *q* will yield $R_0, R_n, f_0, h_0.$ Considering system of equations that consists of polynomials of the form $R_n\log _{{\bar{g}}}D = R_nf_{\lambda }\log _{{\bar{g}}}B - R_0h_{\lambda }\log _{{\bar{g}}}A,$ yields $R_0, R_n, f_{\lambda },$ and $h_{\lambda }$ modulo *q*. Let $\lambda = 3$. One way to determine which elements of the equivalence classes of $R_0, R_n, f_0, f_{\lambda }, h_0$, and $h_{\lambda }$ are the actual solutions in ${\mathbb {Z}}/\varphi (p){\mathbb {Z}}$, is to use brute force search to find coefficients $c_{011\ldots 1}, c_{111\ldots 1}, c_{211\ldots 1}, c_{311\ldots 1}$ and $f_1, f_2, h_1, h_2$ and then compare the expressions $R_0[f_0c_{311\ldots 1}+f_{1}c_{211\ldots 1}+f_2c_{111\ldots 1}+f_3c_{011\ldots 1}]$ and $R_n[h_0c_{311\ldots 1}+h_{1}c_{211\ldots 1}+h_2c_{111\ldots 1}+h_3c_{011\ldots 1}]$ for all $R_0, R_n, f_0, h_0, f_{3}, h_{3}$ in the equivalence classes to the actual values $p_{3111\ldots 1}$ and $q_{311\ldots 1}$ in ${\mathbb {Z}}/\varphi (p){\mathbb {Z}}$. The classical complexity in this case is ${\mathscr {O}}((2^{3x+1})(\varphi (p))^8)$. Otherwise, it is impossible to deterministically lift the solution to ${\mathbb {Z}}/\varphi (p){\mathbb {Z}}$ since these equations can not be considered modulo $2^x$. This approach can yield *A*, *B*, *C* and *D*. The term *E* can be expressed using *A*, *B*, *C*, *D* as $E = A^{Q}B^{-P}C^{-N_0}D^{-N_n}$.

Lastly, we check if the term *E* can be used to gain any private information. The term *E* can be written as

$$\begin{aligned} E = g^{R_0R_n[h(x_0)E_{\phi }(x_0) - f(x_0)E_{\psi }(x_0)]} = B^{R_0E_{\phi }(x_0)}A^{R_nE_{\psi }(x_0)} \text { (mod }p). \end{aligned}$$

Taking logarithm with respect to some generator ${\bar{g}} \in {\mathbb {Z}}/p{\mathbb {Z}}$ yields

$$\begin{aligned} \log _{{\bar{g}}}E = \log _{{\bar{g}}}B(R_0E_{\phi }(x_0)) - \log _{{\bar{g}}}A(R_nE_{\psi }(x_0)). \end{aligned}$$

It is natural to consider a system of such equations for every new $x_0, A, B$ and *E*; however, the unknowns $E_{\phi }(x_0)$ and $E_{\psi }(x_0)$ change with every new choice of $x_0$ so the system regardless of the number of polynomials is always underdetermined. Modulo *q*, the system is also underdetermined and does not produce unique solutions.

Another possible attack to deduce the private key from signature utilizes the public key. We describe it in the following proposition.

#### Proposition 4.12

For any hashed documents or message value $x_0$, cracking the MPPK/DS using signature, obtained from communication records, and public key has classical time complexity of ${\mathscr {O}}([2(2\lambda+1)\log p]q^{\frac{3}{2}}2^{x(n+1)+x/2}+2(\lambda+1) \times 2^{x})$ and quantum complexity of ${\mathscr {O}}(\sqrt{q}\sqrt{2^{x(n+1)}}+2(\lambda+1) \times \sqrt{2^{x}})$.

#### Proof

Start by computing $\log _{{\bar{g}}}A$ and $\log _{{\bar{g}}}B$ modulo *q* for different values of *A* and *B* associated with different $x_0$ to obtain a system of equations of the form

$$\begin{aligned} f'_0 + (f'_1 - \theta h'_1)x_0 + (f'_2 - \theta h'_2)x_0^2 + \ldots + (f'_{\lambda } - \theta h'_{\lambda })x_0^{\lambda } = \theta h'_0 \ \text { (mod } q), \end{aligned}$$

where $$\theta = \frac{\log _{{\bar{g}}}A}{\log _{{\bar{g}}}B},$$ and $$f'_t = R_0f_t, h'_t = R_nh_t$$ for all $$t \in \{0, 1, \ldots , \lambda \}$$. This step can be done in polynomial time using Shor’s algorithm implemented on a quantum computer. However, classically, one needs to use Baby-Step-Giant-Step algorithm with classical computational complexity of $${\mathscr {O}}(2\sqrt{p}\log p)$$ for each value *θ.* Thus, to create the said system of equations the total complexity of this step is $$(2\lambda+1)\,\,\mathcal{O}\,(2\sqrt{p}\log p)$$. Then use brute force search to find the value $$h'_0$$, since the system is homogeneous. Classical complexity of the brute force search is $${\mathscr {O}}(q)$$ and quantum complexity is $${\mathscr {O}}(\sqrt{q})$$ due to Grover’s algorithm. The value $$h'_0$$ can be used to find $$f'_t$$ for all $$t \in \{0, 1, \ldots \lambda \}$$ and $$h'_l$$ for all $$l \in \{1, \ldots , \lambda \}$$. Once these values are found, they can be used to create a matrix modulo *q* with respect to the coefficients of the public polynomials $$P(x_0, x_1, \ldots , x_m)$$ and $$Q(x_0, x_1, \ldots , x_m)$$ as shown in "Security of the private key given the public key". This matrix is used to find the coefficients $$c_{k111\ldots 1}$$ of the base polynomial. Note that the coefficients of the noise functions and the base polynomial can be used to find $$R_0$$ and $$R_n$$, and therefore, $$f_t$$ and $$h_t$$ for $$t \in \{0, 1, \ldots , \lambda \}$$. Everything computed using this approach up to this point is computed modulo *q*. Now, we lift the values $$c_{k11\ldots 1}$$ for $$k \in \{0, 1, \ldots , n\}$$ to the ring $${\mathbb {Z}}/\varphi (p){\mathbb {Z}}$$, and use these values as well as the coefficients of the public polynomial $$P(x_0, x_1, \ldots , x_m)$$ to check if the lift is successful. It is successful if the inverse of the matrix constructed using the coefficients of the base polynomial multiplied by the vector of the coefficients of the public polynomial $$p(x_0, x_1, \ldots , x_m)$$ yields a vector with a few bottom values equal to 0. We discussed this construction in more detail "Security of the private key given the public key". Classical complexity of this lifting part is $${\mathscr {O}}(2^{x(n+1)})$$, and the quantum complexity is $${\mathscr {O}}(\sqrt{2^{x(n+1)}})$$ due to Grover’s algorithm. The lifted values of the base polynomial coefficients are then used to find $$f'_t$$ and $$h'_t$$ in the ring $${\mathbb {Z}}/\varphi (p){\mathbb {Z}}$$ for all $$t \in \{0, 1, \ldots , \lambda \}$$. In order to find $$R_0$$ and $$R_n$$ in the ring $${\mathbb {Z}}/\varphi (p){\mathbb {Z}}$$, one can simply divide the coefficients $$R_0c_{011\ldots 1}$$ of $$N_0$$ by $$c_{011\ldots 1}$$ and $$R_nc_{n11\ldots 1}$$ of $$N_n$$ by $$c_{n11\ldots 1}$$. The only thing left to do in order to be able to create *A*, *B*, *C* and *D* for any $$x_0$$ is to lift $$f_t$$ and $$h_{t}$$ to the ring $${\mathbb {Z}}/\varphi (p){\mathbb {Z}}$$ for all $$t \in \{0, 1, \dots, \lambda\}$$. That can be done by comparing the values $$f'_t$$ and $$h'_{t }$$ computed using matrix of base polynomial coefficients and the values $$R_0$$, $$R_n$$ that are known and $$f_t, h_{t }$$ lifted from $${\mathbb {Z}}/q{\mathbb {Z}}$$. Classical complexity of lifting values $$f_t$$ and $$h_{t}$$ is $${\mathscr 
{O}}(2(\lambda+1)2^{x})$$, and quantum complexity is $${\mathscr {O}}(2(\lambda+1)\sqrt{2^{x}})$$ due to Grover’s algorithm. The overall classical complexity is then $${\mathscr {O}}([2(2\lambda+1)\log p]q^{\frac{3}{2}}2^{x(n+1)+x/2} + 2(\lambda+1)\times 2^x).$$ Quantum complexity is $${\mathscr {O}}(\sqrt{q}\sqrt{2^{x(n+1)}}+2(\lambda+1) \times \sqrt{2^{x}})$$. Using this attack the malicious party can compute *A*, *B*, *C* and *D* for any hashed documents or message value $$x_0.$$ The value *E* can then be expressed as $$E = A^QB^{-P}C^{-N_0}D^{-N_n}.$$

#### Proposition 4.13

For any hashed document or message value $x_0$, cracking the MPPK/DS using only signatures obtained from communication records, has classical time complexity of ${\mathscr {O}}(4(\lambda+1)p^{\lambda +1}[\sqrt{p}\log p]2^{x(2\lambda+4)})$ and quantum complexity of ${\mathscr {O}}(\sqrt{4(\lambda+1)p^{\lambda + 1}2^{x(2\lambda+4)}})$.

#### Proof

This attack utilizes signature components *A*, *B*, *C* and *D* without the public key. Note that for any generator ${\bar{g}} \in {\mathbb {F}}_p$, logarithm $\log _{{\bar{g}}}A = [f'_0 + f'_1x_0 + \ldots + f'_{\lambda }x_0^{\lambda }]\log _{{\bar{g}}}g.$ This equation has $\lambda + 1$ unknowns $f'_t = R_0f_t$, for $t \in \{0, \ldots , \lambda \}$. Let an adversary consider the following system of equations, where values *A*_*k*_ are obtained from communication records between the signer and the verifier for all values $k \in \{1, \dots, \lambda+1\}$. 

18 $$\begin{aligned}{}&\log _{{\bar{g}}}A_1 = [f'_0 + f'_1x_0 + \ldots + f'_{\lambda }x_0^{\lambda }]\log _{{\bar{g}}}g_1\nonumber \\&\log _{{\bar{g}}}A_2 = [f'_0 + f'_1\hat{x_0} + \ldots + f'_{\lambda }\hat{x_0}^{\lambda }]\log _{{\bar{g}}}g_2\nonumber \\&\vdots \nonumber \\&\log _{{\bar{g}}}A_{\lambda +1} = [f'_0 + f'_1\tilde{x_0} + \ldots + f'_{\lambda }\tilde{x_0}^{\lambda }]\log _{{\bar{g}}}g_{\lambda +1}. \end{aligned}$$

Classical computational complexity of computing discrete logarithms $\log _{{\bar{g}}}A_{k}$ is ${\mathscr {O}}(\sqrt{p}\log p)$ for any $k \in \{1, \ldots , \lambda +1\}$, using Baby-Step-Giant-Step algorithm. On the other hand, using quantum computer one can compute discrete logarithms in polynomial time. The malicious party can brute force search for values $\log _{{\bar{g}}}g_k$ for all $k \in \{1, \ldots , \lambda +1\}.$ The complexity of this search is ${\mathscr {O}}(p^{\lambda +1}).$ Once, these values are found, the adversary can solve deterministically the system of equations modulo *q* in the Eq. (18) to retrieve private information $f'_t$ for $t \in \{0, \ldots , \lambda \}$. The same values $\log _{{\bar{g}}}g_k$, for all $k \in \{1, \ldots , \lambda +1\}$, can be used to deterministically find the private information $h'_t = R_nh_t$, for $t \in \{1, \ldots , \lambda \}$, from the system of equations similar to the one in Eq. (18) with signature component *B *computed modulo *q*. Classical complexity of this part comes from solving discrete logarithms of the form $\log _{{\bar{g}}}B_{k}$ for all $k \in \{1, \ldots , \lambda +1\}$. The complexity is equal to ${\mathscr {O}}([\lambda +1][\sqrt{p}\log p]).$ Using private values found this way, the adversary can construct *A* and *B* for any message. For values *C* and *D* the adversary can consider the following system of equations modulo *q*

19 $$\begin{aligned}{}&\log _{{\bar{g}}}C_1 = [c_0 + c_1x_0 + \ldots + c_{\lambda }x_0^{\lambda }]\log _{{\bar{g}}}g_1\nonumber \\&\log _{{\bar{g}}}C_2 = [c_0 + c_1\hat{x_0} + \ldots + c_{\lambda }\hat{x_0}^{\lambda }]\log _{{\bar{g}}}g_2\nonumber \\&\vdots \nonumber \\&\log _{{\bar{g}}}C_{\lambda +1} = [c_0 + c_1\tilde{x_0} + \ldots + c_{\lambda }\tilde{x_0}^{\lambda }]\log _{{\bar{g}}}g_{\lambda +1}, \end{aligned}$$

where $s_0(x_0) = c_0 + c_1x_0 + \ldots + c_{\lambda }x_0^{\lambda }$ for $s_0(x_0)$ as described in "Signing algorithm". Values $\log _{{\bar{g}}}g_k$, for all $k \in \{1, \ldots , \lambda +1\}$ are known so the complexity comes from solving discreet logarithms $\log _{{\bar{g}}}C_k$ for $k \in \{1, \ldots , \lambda +1\}.$ Similar system of equations can be created for the signature component *D*. All the obtained private values need to be lifted to the ring ℤ/φ(p)ℤ. The classical complexity of the lifting step is (2^*x*^^(^^2^^λ^^+^^4^^)^).The component *E* can be calculated using $E = A^{Q}B^{-P}C^{-N_0}D^{-N_n}.$ Overall, the total classical complexity of the attack is ${\mathscr {O}}(p^{\lambda +1}4(\lambda +1)(\sqrt{p}\log p)2^{x(2\lambda+4)}).$ Quantum complexity is ${\mathscr {O}}(\sqrt{4(\lambda+1)p^{\lambda +1}2^{x(2\lambda+4)}})$ using Grover’s algorithm for brute force search.

We conclude that the smallest computational complexity of finding private key from signature, depending on the parameter choices, is either ${\mathscr {O}}([2(2\lambda+1)\log p]q^{\frac{3}{2}}2^{x(n+1)+x/2}+2(\lambda+1)\times 2^x)$ using a classical computer, and ${\mathscr {O}}(\sqrt{q}\sqrt{2^{x(n+1)}}+2(\lambda+1)\times\sqrt{2^{x}})$ using a quantum device.

### Spoofing attacks

Recall, that the base $g \in {\mathbb {F}}_p$, as well as polynomials $f(x_0), h(x_0) \in {\mathbb {Z}}/(\varphi (p)){\mathbb {Z}}[x]$, constants $R_0, R_n \in {\mathbb {Z}}/\varphi (p){\mathbb {Z}}$, and polynomials $E_{\phi }(x_0), E_{\psi }(x_0) \in {\mathbb {Z}}/(\varphi (p)){\mathbb {Z}}[x]$ are unknown. The attacker might try to look at any existing relationship between the values *A*, *B*, *C*, *D* and *E*. Then, if any other values $A' \ne A, B' \ne B, C'\ne C, D' \ne D$ and $E'\ne E$ satisfy the same relationship, they might be used as a signature, and pass verification. We showed in the “Security of the private key given the public key” section that none of the values *A*, *B*, *C*, *D* and *E* can be expressed in terms of one another.

Another way for the malicious party to carry out a spoofing attack is to break the value $A = g^{R_0f(x_0)}$ into

$$\begin{aligned} (g^{R_0f_0}) \times (g^{R_0f_1})^{x_0}+ (g^{R_0f_2})^{x_0^2}+ \cdots + (g^{R_0f_{\lambda }})^{x_0^{\lambda }} \end{aligned}$$

and obtain every element of the form $g^{R_0f_i}$ for $i \in \{0, \ldots , \lambda \}$. Similarly, obtain the terms $g^{R_nh_i}$ from $B=g^{R_nh(x_0)}$, the terms $g^{R_nf_0h_i}, g^{-R_nh_0f_i}$ from $C = g^{s_0}$, and terms $g^{R_0f_{\lambda }h_i}, g^{-R_0h_{\lambda }f_i}$ from $D = g^{s_n}$ for all $i \in \{0, \ldots , \lambda \}$. The attacker also need to obtain the terms $g^{R_0R_n[h_ie_{\phi _{j}}-f_ie_{\psi _{j}}]}$ from *E*. This way, the attacker can easily change the original document $x_0$ into a different document with the correct signature, in other words, achieve universal forgery. In this case, the verifier will not be able to determine any malicious activity as the document and the signature will pass the verification without raising any issues.

We show that such an attack is not applicable because it does not yield deterministic results if the terms described above are found using brute force.

#### Proposition 4.14

Generating all components of the form $g^{R_0f_i}$, $g^{R_nh_i}$, $(g^{R_nf_0h_i}, g^{-R_nh_0f_i}),$ $(g^{R_0f_{\lambda }h_i}, g^{-R_0h_{\lambda }f_i}),$ and $g^{R_0R_n[h_ie_{\phi _{j}}-f_ie_{\psi _{j}}]}$ associated with *A*, *B*, *C*, *D* and *E* respectively for each $i \in \{0, 1, \ldots , \lambda \}$, has time complexity of ${\mathscr {O}}(p^{n+4\lambda })$. Once the correct tuples are found, they can be used to repeatedly forge a signature for any hashed message $x_0.$

#### Proof

For the proof we consider a simplified example with quadratic polynomials $f(x_0)$ and $h(x_0)$. The proof for the general case is identical. We have the following equations

$$\begin{aligned} A= \,& {} g^{R_0f(x_0)} = (g^{R_0f_0}) \times (g^{R_0f_1})^{x_0} \times (g^{R_0f_2})^{x_0^2} \ (\text { mod }p) \text{ and } \\ B=\, & {} g^{R_nh(x_0)} = (g^{R_nh_0}) \times (g^{R_nh_1})^{x_0}\times (g^{R_nh_2})^{x_0^2} \ (\text { mod }p). \end{aligned}$$

Using brute force, the attacker needs to go over every term $g^{R_0f_1}$ and $g^{R_0f_2}$, and consider an equality of the form

$$\begin{aligned} (g^{R_0f_1})^{x_0} \times (g^{R_0f_2})^{x_0^2} = \frac{A}{g^{R_0f_0}} \ (\text { mod }p) \end{aligned}$$

which yields $g^{R_0f_0}.$ Thus, to generate tuples $(g^{R_0f_0}, g^{R_0f_1}, g^{R_0f_2})$ the malicious party needs to sample $p^2$ elements. Since the ratios of the form $\frac{R_0f_i}{R_nh_i}$ do not exist for any $i \in \{0,1, 2\},$ the attacker has to find tuples

$$\begin{aligned} (g^{R_nh_0}, g^{R_nh_1}, g^{R_nh_2}) \end{aligned}$$

using the same strategy. However, the terms $g^{R_0f_1}$ and $g^{R_0f_2}$ are simply elements of the field ${\mathbb {F}}_{p_s}$. The attacker has already calculated $a^{x_0}b^{x_0^2}$ for all possible elements $a,b \in {\mathbb {F}}_{p_s}$. All such terms can be reused to find all possible $g^{R_nh_0}$ from the equality

$$\begin{aligned} a^{x_0}b^{x_0^2} = \frac{B}{g^{R_nh_0}} \ (\text { mod 
}p). \end{aligned}$$

Thus, to construct tuples $(g^{R_nh_0}, g^{R_nh_1}, g^{R_nh_2})$ the attacker does not need to sample any more terms but the calculations require going through $p^2$ terms. Similarly, the existing sampled terms $a^{x_0}b^{x_0^2}$ for all possible elements $a,b \in {\mathbb {F}}_{p_s}$ can be reused for *C*. Indeed, the equations are

$$\begin{aligned} C&= g^{R_nf_0h_0 - R_nf_0h_0} \times (g^{R_nf_0h_1})^{x_0} \times (g^{R_nf_0h_2})^{x_0^2} \times (g^{-R_nh_0f_1})^{x_0} \times (g^{-R_nh_0f_2})^{x_0^2} \ (\text { mod }p)\\&= (g^{R_nf_0h_1})^{x_0} \times (g^{R_nf_0h_2})^{x_0^2} \times (g^{-R_nh_0f_1})^{x_0} \times (g^{-R_nh_0f_2})^{x_0^2} \ (\text { mod }p)\\&= (g^{R_nf_0h_1-R_nh_0f_1})^{x_0} \times (g^{R_nf_0h_2-R_nh_0f_2})^{x_0^2} \ (\text { mod }p). \end{aligned}$$

Checking the value for *C* requires going through $p^2$ items. In our example, with quadratic functions $f(x_0), h(x_0)$, the equation for *D* has the following form

$$\begin{aligned} D&= (g^{R_0[h_2f_2-f_2h_2]})^{x_0^2}\times (g^{R_0h_0f_2})\times (g^{R_0h_1f_2})^{x_0}\times (g^{-R_0f_0h_2})\times (g^{-R_0f_1h_2})^{x_0} \ (\text { mod }p)\\&= (g^{R_0h_0f_2})\times (g^{R_0h_1f_2})^{x_0}\times (g^{-R_0f_0h_2})\times (g^{-R_0f_1h_2})^{x_0} \ (\text { mod }p)\\&= g^{R_0h_0f_2-R_0f_0h_2}\times (g^{R_0h_1f_2 - R_0f_1h_2})^{x_0} \ (\text { mod }p). \end{aligned}$$

The attacker needs to go through $p^2$ values to check for *D*.

Lastly, the malicious party has to consider the following equation with $\text { mod }p$

$$\begin{aligned} E&= g^{[R_0R_n(h_0e_{\phi _1} - f_0e_{\psi _1})]x_0} \times g^{[R_0R_n(h_0e_{\phi _2} + h_1e_{\phi _1} - f_0e_{\psi _2} - f_1e_{\psi _1})]x_0^2} \\&\quad \times \cdots \times g^{[R_0R_n(h_0e_{\phi _{n-1}} + \ldots + h_2e_{\phi _{n-3}} - f_0e_{\psi _{n-1}} - \ldots - f_2e_{\psi _{n-3}})]x_0^{n+1}} \ \end{aligned}$$

The attacker will need to go through $p^{n}$ elements. One of the terms can be derived as a ration between *E* and the remaining terms of the form $g^{R_0R_n(\sum h_ie_{\phi _j} - f_ie_{\psi _j})}$. The complexity of generating all the tuples is, therefore, ${\mathscr {O}}(p^{n+8})$ for this example. However, there is no efficient way to determine which five tuples, one for each of *A*, *B*, *C*, *D* and *E*, are the correct ones used by the signing party. For that one might try to create *A*, *B*, *C*, *D* and *E* for different $x_0$ and verify that $A^Q = B^PC^{N_0}D^{N_n}$ for different $P, Q, N_0$ and $N_n.$ In the case that the attacker finds the correct tuples associated with *A*, *B*, *C*, *D*, and *E*, since the tuples are independent of $x_0$, the attacker can use them to forge a signature for any $x_0.$ $\square$

There are other ways to carry out a spoofing attack. However, we claim that the following approach is the most efficient.

#### Proposition 4.15

Search for the values *A*, *B*, *C*, *D*, *E*, such that $A^{{\bar{Q}}} = B^{{\bar{P}}}C^{\bar{N_0}}D^{\bar{N_n}}E$ holds for any ${\bar{P}}, {\bar{Q}}, \bar{N_0},$ and $\bar{N_n}$, has time complexity ${\mathscr {O}}(p^{4+m})$ using a classical computer, and ${\mathscr {O}}(\sqrt{p^{4+m}})$ using a quantum computer, where *m* is the number of noise variables.

#### Proof

For each given $x_0$ begin by fixing a choice of values $r_1, \ldots , r_m$ for variables $x_1, \ldots , x_m$. Use such choice of noise values to calculate values of the polynomials ${\bar{P}} = P(x_0, r_1, \ldots , r_m), {\bar{Q}} = Q(x_0, r_1, \ldots , r_m), \bar{N_0} = N_0(r_1, \ldots , r_m),$ and $\bar{N_n} = N_n(x_0, r_1, \ldots , r_m).$ Recall, that $A^{Q} = B^{P}C^{N_0}D^{N_n}E$ for any $P, Q, N_0, N_n$ computed using publicly available coefficients provided by the signer. Thus, given ${\bar{P}}, {\bar{Q}}, \bar{N_0},$ and $\bar{N_n}$ the attacker should look for values *A*, *B*, *C*, *D* and compute $E = A^{{\bar{Q}}}B^{-{\bar{P}}}C^{-\bar{N_0}}D^{-\bar{N_n}}$. To check if the choice of *A*, *B*, *C*,  and *D* is correct, the attacker can use these values and new values ${\bar{P}}, {\bar{Q}}, \bar{N_0}, \bar{N_n}$ to check if $E = A^{Q} B^{-{\bar{P}}}C^{-\bar{N_0}}D^{-\bar{N_n}}$ remains true. If not, discard of the value *A*, *B*, *C*, *D* and look for new ones. There are in total $p^4$ tuples of the form (*A*, *B*, *C*, *D*). Thus, the attacker in the worst case will have to go through all $p^4$ such sets for two fixed ${\bar{P}}, {\bar{Q}}, \bar{N_0},$ and $\bar{N_n}$ until the equality of the form $E = A^{Q}B^{-{\bar{P}}}C^{-\bar{N_0}}D^{-\bar{N_n}}$ is achieved for at least two distinct choices of values ${\bar{P}}, {\bar{Q}}, \bar{N_0}, \bar{N_n}$. Once the attacker finds values *A*, *B*, *C*, *D*, *E* such the equality holds for two values of public polynomials ${\bar{P}}, {\bar{Q}}, \bar{N_0}, \bar{N_n}$, they will have to check whether the equality holds for all the other choices of $r_1, \ldots , r_m$. If so, then the values *A*, *B*, *C*, *D*, *E* can be used by the attacker as the signature, and will result in the universal forgery. The overall complexity of this attack is ${\mathscr {O}}(p^{4+m})$ using a classical computer.

#### Corollary 4.16

The most efficient spoofing attack has classical complexity ${\mathscr {O}}(p^{4+m})$ and quantum complexity ${\mathscr {O}}(\sqrt{p^{4+m}})$, where *m* is the number of noise variables.

### Security conclusion

The best classical complexities of universal forgery of the signature are as follows. [*Attack 1*] The attacker can use public key to crack for the private key and create signature $\{A, B, C, D, E\}$ for any message or document. The classical complexity of this attack is ${\mathscr {C}}_1 = {\mathscr {O}}(\varphi (p)^{n+1}+2 \times 2^{x(\lambda+1)})$. [*Attack 2*] Another attack on the public key that we have discovered has classical complexity of ${\mathscr {C}}_2 = {\mathscr {O}}(q^{\lambda+2}[2\left\lceil \frac{n+1} {\lambda+2}\right\rceil 2^{x(\lambda+2)}])$. *[Attack 3]* A different attack the malicious party can undertake is to gain enough information from a genuine signature obtained from a communication interception between the signer and verifier as well as the public key and use that information to recreate a full signature for any message or document $x_0$. The classical complexity of this attack is ${\mathscr {C}}_3 = {\mathscr {O}}([2(2\lambda+1)\log p]q^{\frac{3}{2}}2^{x(n+1)+x/2}+2(\lambda+1)\times 2^x)$. [*Attack 4*] A similar attack that only uses a genuine signature has classical complexity of ${\mathscr {C}}_4 = {\mathscr {O}}(4(\lambda+1)p^{\lambda +1}[\sqrt{p}\log p ]2^{x(2\lambda+4)})$. [*Attack 5*] And lastly, the attacker can directly spoof the signature. The complexity of direct spoofing is ${\mathscr {C}}_5 = {\mathscr {O}}(p^{4+m}).$ Of these five attacks, the attack that use genuine signatures is in favor of the attacker with classical complexity ${\mathscr {C}}_2 = {\mathscr {O}}([2(2\lambda+1)\log p]q^{\frac{3}{2}}2^{x(n+1)+x/2}+2(\lambda+1)\times 2^x).$

For complexities of cracking MPPK/DS using a quantum computer, the adversary can use public key only attack that has quantum complexity of ${\mathscr {C}}_1 = {\mathscr {O}}(\sqrt{\varphi (p)^{n+1}} + 2 \times \sqrt{2^{x(\lambda+1)}}).$ Another attack that uses public keys has quantum complexity of ${\mathscr {C}}_2 = {\mathscr {O}}(\sqrt{q^{\lambda+2} [2\left\lceil \frac{n+1} {\lambda+2}\right\rceil 2^{x(\lambda+2)}]})$. The adversary can also use honest signatures obtained from communication records. The attack that uses honest signatures in conjunction with public keys has quantum complexity of ${\mathscr {C}}_3 = {\mathscr {O}}(\sqrt{q}\sqrt{2^{x(n+1)}}+2(\lambda+1)\times\sqrt{2^{x}})$. The attack that uses signatures only has quantum complexity ${\mathscr {C}}_4 = {\mathscr {O}}(\sqrt{4(\lambda+1)p^{\lambda +1}2^{x(2\lambda+4)}}).$ Lastly, the attacker can directly spoof the signature. Quantum complexity of this case is ${\mathscr {C}}_4 = {\mathscr {O}}(\sqrt{p^{4+m}}).$

## Brief benchmarking results and optimal parameters of MPPK/DS

We now introduce optimal parameters and report benchmarking results for MPPK/DS. For benchmarking. we used the NIST recognized SUPERCOP benchmarking tool. The SUPERCOP was run on a 16-core Intel®Core™i7-10700 CPU at 2.90 GHz system.

### Configuration

We begin by requiring that the prime *p* is a generalized safe prime (or a special Cullen prime) such that $p = 2^xq+1$, where *q* is a prime number. We will further discuss *x* and *q* with respect to the desirable security level. We require that noise coefficients $R_0$ and $R_n$ are even non-zero numbers in the ring ${\mathbb {Z}}/\varphi (p){\mathbb {Z}}.$ We require that *A*, *B*, *C*, *D*,  and *E* are all integers in the field ${\mathbb {F}}_p$ not equal to 0 or 1. We require that neither $N_0$ nor $N_n$ are equal to zero modulo $\varphi (p).$

Note that the smallest classical complexity is ${\mathscr {C}}_3 = {\mathscr {O}}([2(2\lambda+1)\log p]q^{\frac{3}{2}}2^{x(n+1)+x/2}+2(\lambda+1)\times 2^x)$, which depends majorly on *x*. Thus, when making decisions about *x* and *q*, it is important to make *x* and *q* sufficiently large to guarantee the security of the DS scheme. We also suggest to set $m \ge 1, n \ge 2,$ and $\lambda \ge 2$ for optimal performance of key generation, signing, and verification to achieve the NIST security three levels.

We provide optimal parameters for each security level, considering classical complexity of each attack we have discovered in Table 2. That is, the parameters given in Table 2 are sufficient to meet corresponding NIST security level and avert the corresponding attack.

**Table 2:** Proposed MPPK/DS configurations to meet corresponding NIST Security level and avert corresponding attack, with values given as $(\log _2q, x, \log _2p, n, \lambda , m)$.

Attack	Complexity	Security level		
		Level I	Level III	Level V
1	${\mathscr {C}}_1 = {\mathscr {O}}(\varphi(p)^{n+1}+2\times 2^{x(\lambda+1)})$	(32, 32, 64, 2, 2, 1)	(32, 32, 64, 3, 2, 1)	(32, 32, 64, 4, 2, 1)
2	${\mathscr {C}}_2 = {\mathscr {O}}({q^{\lambda+2} [2\lceil \frac{n+1} {\lambda+2}\rceil 2^{x(\lambda+2)}]})$	(32, 32, 64, 2, 1, 1)	(32, 32, 64, 2, 2, 1)	(32, 32, 64, 2, 3, 1)
3	${\mathscr {C}}_3 = {\mathscr {O}}([2(2\lambda+1)\log p]q^{\frac{3}{2}}2^{x(n+1)+x/2}+2(\lambda+1)\times 2^x)$	(32, 32, 64, 2, 2, 1)	(32, 32, 64, 4, 2, 1)	(32, 32, 64, 6, 2, 1)
4	${\mathscr {C}}_4 = {\mathscr {O}}(4(\lambda+1)p^{\lambda+1}[\sqrt{p}\log p]2^{x(2\lambda+4)})$	(32, 32, 64, 2, 2, 1)	(32, 32, 64, 2, 2, 1)	(32, 32, 64, 2, 3, 1)
5	${\mathscr {C}}_5 = {\mathscr {O}}(p^{4+m})$	(32, 32, 64, 2, 2, 1)	(32, 32, 64, 2, 2, 1)	(32, 32, 64, 2, 2, 1)
1–5	${\mathscr {C}}_1, {\mathscr {C}}_2, {\mathscr {C}}_3, {\mathscr {C}}_4, {\mathscr {C}}_5$^a^	(32, 32, 64, 2, 2, 3)	(32, 32, 64, 4, 2, 3)	(32, 32, 64, 6, 3, 2)

^a^All the classical complexity estimations considered together.

### Benchmarking results

Assuming the parameters shown in Table 2 for each corresponding security level and complexity of all attacks considered together, that is the last row of Table 2, we report benchmarking results about MPPK/DS. We used the NIST accepted SUPERCOP benchmarking tool. All the NIST third round finalists’ SUPERCOP measurement data was contributed to SUPERCOP. Thus, we take advantage of the common performance measurement platform and report on the benchmarking results of MPPK/DS alongside the NIST third-round DS finalists, namely Crystals-Dilithium, Falcon, and Rainbow algorithms. The system used for all primitives is a 16-core Intel®Core™i7-10700 CPU at 2.90 GHz.

For this paper, we use a snapshot of detailed data reported separately^62^. Performance measurements presented in this section are median values. The average values, quartile values, as well as standard deviation, and error rates are available separately^62^.

We first present the reader with Table 3, illustrating public key sizes and signature sizes of the MPPK/DS scheme and the NIST third round finalists in bytes, for NIST security levels I, III, and V. Public key sizes of the MPPK/DS algorithm are calculated using the formula $m[2(n+\lambda -1)+2] = 2m(n+\lambda )$ over *GF*(*p*), since public key consists of the coefficients of polynomials $P(x_0, x_1, \ldots , x_m)$ and $Q(x_0, x_1, \ldots , x_m)$, each having $n+\lambda -1$ coefficients for each choice of noise variables, and coefficients of the noise functions $N_0(x_1, \ldots , x_m), N_n(x_0, x_1, \ldots , x_m)$ with one coefficient each, for every choice of noise variables. Finite field being 64 bits for all three security levels, result in public key sizes of 192, 288,  and 288 bytes for levels I, III and V respectively. Considering together with the NIST third round finalists, MPPK/DS offers rather small public key sizes.

**Table 3:** Public Key and Signature sizes of the the MPPK/DS scheme as well as the NIST PQC Round 3 Finalists, with values given in Bytes corresponding to various NIST Security Levels.

Signature	Public key size (B)			Signature size (B)		
Scheme	I	III	V	I	III	V
MPPK/DS	192	288	288	80	120	160
Rainbow^a^	161,600	882,080	1,930,600	66	164	212
Dilithium^b^	–	1952	2592	–	3293	4,595
Falcon^c^	897	–	1793	690	–	1330

^a^The *rainbow1aclassic363232* primitive was measured for Level I, *rainbow3cclassic683248* for Level III, and *rainbow5cclassic963664* for Level V.

^b^Dilithium does not provide primitive for NIST Level I, *dilithium3* was used for Level III, and *dilithium5* for Level V.

^c^For Falcon, *falcon512dyn* was measured for Level I, no primitive was measured for Level III, *falcon1024dyn* was measured for Level V.

Recall, that there are five components in the signature, namely (*A*, *B*, *C*, *D*, *E*). Each such signature element should be of sufficient size to prevent brute force attacks, leading to spoofing. For level I, therefore, each component of the signature element is 128 bits. The entire signature is $5 \times 128= 640$ bits, which is 80 bytes. Similarly, the signature size for level III is 120 bytes, and 160 bytes for level V. Based on values in Table 3, sizes of the MPPK/DS are comparable and some cases noticeably smaller than the corresponding signature sizes of the three NIST finalists.

Key generation performance comparison between the MPPK/DS scheme and the NIST finalists is given in Table 4. From the data shown in the table, MPPK/DS offers efficient key generation, outperforming the NIST Round 3 finalists. A similar account is observed for the signing procedure. Note that the values given in both tables are median values of the SUPERCOP measurement.

**Table 4:** Median values given in clock cycles, corresponding to the Performance measurement of the MPPK/DS scheme as well as the NIST PQC Round 3 Finalists for various NIST Security Levels.

Security level	Level I	Level III	Level V
**Key generation**			
MPPK/DS	22,437	36,700	47,668
Rainbow^a^	20,788,655	123,007,216	263,207,040
Dilithium^b^	–	322,993	454,373
Falcon^c^	32,557,525	–	91,533,955
**Signing procedure**			
MPPK/DS	42,286	57,223	63,534
Rainbow^a^	180,675	898,223	1,491,838
Dilithium^b^	–	1,163,882	1,041,113
Falcon^c^	10,268,556	–	22,499,756
**Signature verification procedure**			
MPPK/DS	48,965	75,980	87,567
Rainbow^a^	21,258	177,094	332,196
Dilithium^b^	–	313,009	482,670
Falcon^c^	68,858	–	138,492

^a^The *rainbow1aclassic363232* primitive was measured for Level I, *rainbow3cclassic683248* for Level III, and *rainbow5cclassic963664* for Level V.

^b^Dilithium does not provide primitive for NIST Level I, *dilithium3* was used for Level III, and *dilithium5* for Level V.

^c^For Falcon, *falcon512dyn* was measured for Level I, no primitive was measured for Level III, *falcon1024dyn* was measured for Level V.

Table 4 also depicts the median values of MPPK/DS and NIST Round 3 finalists’ signature verification performance in clock cycles. The data in the table demonstrates that the signature verification performance of the MPPK/DS primitive is comparable to the Rainbow signature scheme and faster than the Crystals-Dilithium as well as the Falcon algorithms.

The reader will notice that the overall performance of the MPPK/DS scheme is more comparable to the Rainbow scheme than other NIST Round 3 finalists. To explore this a little further, we include Tables 5 and 6 to compare the public key and signature sizes, as well as the performance of the MPPK/DS algorithm and the NIST Round 3 multivariate finalist and alternative algorithms, Rainbow and GeMSS^17,31^.

**Table 5:** Public Key Sizes of the the MPPK/DS scheme as well as the NIST PQC Round 3 multivariate DS schemes, with values given in Bytes.

Signature	Public key size (B)			Signature size (B)		
Scheme	I	III	V	I	III	V
MPPK/DS	192	288	288	80	120	160
Rainbow^a^	161, 600	882, 080	1, 930, 600	66	164	212
GeMSS^b^	352, 188	1, 237, 964	3, 040, 700	32.25	51.375	72

^a^The *rainbow1aclassic363232* primitive was measured for Level I, *rainbow3cclassic683248* for Level III, and *rainbow5cclassic963664* for Level V.

^b^*GeMSS128* primitive corresponds to values for level I, *GeMSS192* corresponds to values for level III, and *GeMSS256* corresponds to values for level V.

**Table 6:** Performance of the the MPPK/DS scheme as well as the NIST PQC Round 3 multivariate DS schemes, with values given in clock cycles.

Primitive	Level I	Level III	Level V
**Key generation**			
MPPK/DS	22,437	36,700	47, 668
Rainbow^a^	20,788,655	123,007,216	263,207,040
GeMSS^b^	36,800,000	167,000,000	508,000,000
**Signing procedure**			
MPPK/DS	42,286	57,223	63,534
Rainbow^a^	180,675	898,223	1,491,838
GeMSS^b^	529,000,000	1720,000,000	2830,000,000
**Signature Verification procedure**			
MPPK/DS	48,965	75,980	87,567
Rainbow^a^	21,258	177,094	332,196
GeMSS^b^	84,600	233,000	550,000

^a^The *rainbow1aclassic363232* primitive was measured for Level I, *rainbow3cclassic683248* for Level III, and *rainbow5cclassic963664* for Level V.

^b^*GeMSS128* primitive corresponds to values for level I, *GeMSS192* corresponds to values for level III, and *GeMSS256* corresponds to values for level V.

Table 5 shows that public key sizes of the MPPK/DS are noticeably smaller than public key sizes of other multivariate primitives considered. However, signature sizes of the MPPK/DS are greater than those of the GeMSS algorithm and comparable to the Rainbow algorithm.

Table 6 provides comparison of the performance measurements between MPPK/DS, and Rainbow and GeMSS signature schemes. All the values are given in clock cycles. Note, however, that the values for MPPK/DS and Rainbow are taken from our own benchmarking work, using SUPERCOP and only the median value are provided in the table. The system that was used to measure the performance of MPPK/DS and Rainbow is a 16-core Intel®Core™i7-10700 CPU at 2.90 GHz. On the other hand, the values for GeMSS were taken from their official online page, The performance was measured using MQsoft using Skylake processor Intel®Core™i7-6600U CPU at 2.60GHz.

Table 6 values show that MPPK/DS achieve more efficient key generation and signature creation procedures compared to the Rainbow and GeMSS signature schemes. However, the signature verification performance of MPPK/DS is not as efficient as the Rainbow algorithm for level I security. For level III, MPPK/DS performance is comparable with Rainbow and GeMSS. For level V, a noticeable difference between values is observed, with MPPK/DS outperforming both the Rainbow and GeMSS signature schemes.

Overall, MPPK/DS achieves rather small public key and signature sizes and offers efficient key generation, signature creation, and signature verification procedures compared to other PQC signature schemes.

## Conclusion

We presented a new quantum-safe digital signature algorithm called MPPK/DS. It is based on the Kuang et al.’s MPPK KEM algorithm. MPPK/DS is a multivariate, quantum-safe and falls into the category of probabilistic DS algorithms. Indeed, verifying the same signature multiple times with different noise variable values meets the same verification relationship. The core of the signing-verifying relationship is a modular arithmetic property that given *x* co-prime to *n* and two integers *a* and *b* such that $a \equiv b \ (\text {mod }\varphi (n)), \text { then } x^a = x^b \ (\text {mod }n),$ where $\varphi (n)$ is the Euler’s totient function evaluated at *n*. Using a generalized safe prime $p=2^xq + 1$, discussed in "MPPK digital signature and verification", we performed security analysis for the MPPK/DS algorithm to conclude that the complexity of the best possible attack on the MPPK/DS is ${\mathscr {O}}([2(2\lambda+1)\log p]q^{\frac{3}{2}}2^{x(n+1)+x/2}+2(\lambda+1)\times 2^x)$ using classical computing, and ${\mathscr {O}}(\sqrt{q}\sqrt{2^{x(n+1)}}+2(\lambda+1)\times \sqrt{2^{x}})$ and for quantum computing.

We also report briefly on the performance of MPPK/DS measured using the NIST recognized benchmarking toolkit SUPERCOP. The overall performance for key generation, signing, and verifying, is very efficient, outperforming the NIST 3rd round finalists. We provide a detailed performance analysis of the MPPK/DS algorithm in a companion paper^62^. A MPPK/DS implementation is available online^63^.

## Data Availability

All data generated or analysed during this study are included in this published article (and its Supplementary Information files).
